# The Inhibitory Effect of GlmU Acetyltransferase Inhibitor TPSA on *Mycobacterium tuberculosis* May Be Affected Due to Its Methylation by Methyltransferase Rv0560c

**DOI:** 10.3389/fcimb.2019.00251

**Published:** 2019-07-17

**Authors:** Changming Chen, Xiuyan Han, Qiulong Yan, Chao Wang, Liqiu Jia, Ayaz Taj, Lizhe Zhao, Yufang Ma

**Affiliations:** ^1^Department of Biochemistry and Molecular Biology, College of Basic Medical Sciences, Dalian Medical University, Dalian, China; ^2^Department of Microbiology, College of Basic Medical Sciences, Dalian Medical University, Dalian, China; ^3^College of Pharmacy, Dalian Medical University, Dalian, China

**Keywords:** *Mycobacteria tuberculosis*, UDP-GlcNAc, GlmU acetyltransferase, inhibitor, methyltransferases

## Abstract

*Mycobacterium tuberculosis* bifunctional enzyme GlmU is a novel target for anti-TB drugs and is involved in glycosyl donor UDP-N-acetylglucosamine biosynthesis. Here, we found that TPSA (2-[5-(2-{[4-(2-thienyl)-2-pyrimidinyl]sulfanyl}acetyl)-2-thienyl]acetic acid) was a novel inhibitor for GlmU acetyltransferase activity (IC_50_: 5.3 μM). The interaction sites of GlmU and TPSA by molecular docking were confirmed by site-directed mutagenesis. TPSA showed an inhibitory effect on Mtb H37Ra growth and intracellular H37Ra in macrophage cells (MIC: 66.5 μM). To investigate why TPSA at a higher concentration (66.5 μM) was able to inhibit H37Ra growth, proteome and transcriptome of H37Ra treated with TPSA were analyzed. The expression of two methyltransferases MRA_0565 (Rv0558) and MRA_0567 (Rv0560c) were markedly increased. TPSA was pre-incubated with purified Rv0558 and Rv0560c in the presence of S-adenosylmethionine (methyl donor) respectively, resulting in its decreased inhibitory effect of GlmU on acetyltransferase activity. The inhibition of TPSA on growth of H37Ra with overexpressed Rv0558 and Rv0560c was reduced. These implied that methyltransferases could modify TPSA. The methylation of TPSA catalyzed by Rv0560c was subsequently confirmed by LC-MS. Therefore, TPSA as a GlmU acetyltransferase activity inhibitor may offer a structural basis for new anti-tuberculosis drugs. TPSA needs to be modified further by some groups to prevent its methylation by methyltransferases.

## Introduction

Tuberculosis (TB) has existed for millennia, remains a major health problem worldwide (World Health Organization, 2018) and is one of the top ten causes of death all over the world (Mccullough and Lehrer, 2018). More seriously, the increasing emergence of multidrug-resistant (MDR) and extensively drug-resistant (XDR) tuberculosis along with human immunodeficiency virus (HIV) infection presents a serious challenge to control TB effectively (Zhang and Yew, 2015; Blondiaux et al., 2017). For example, in 2017, there were 558,000 new cases with resistance to rifampicin (RFP, the most effective first-line drug) and 82% of new cases were MDR-TB (World Health Organization, 2018). As a consequence, it is a matter of urgency to find more effective candidates of novel anti-tuberculosis drugs.

**Figure 1:**
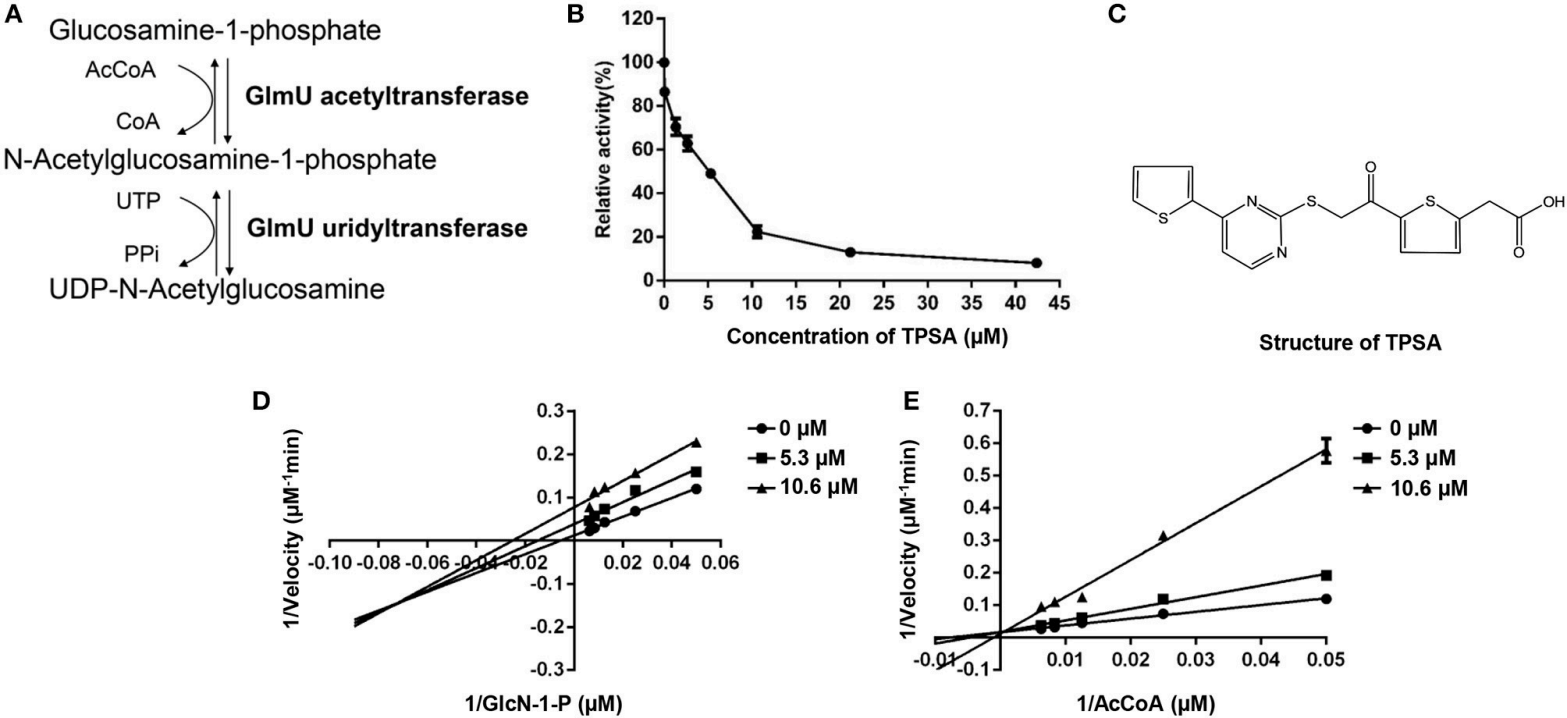
The enzymatic reactions catalyzed by GlmU **(A)** and the inhibition effect of TPSA on GlmU acetyltransferase activity **(B)**, molecular structure of TPSA **(C)**, and the inhibition types of TPSA on GlmU acetyltransferase **(D,E)**. Double reciprocal plots showed 1/v vs. 1/s, where v was the initial velocity and s was the substrate concentration for GlcN-1-P and AcCoA. In **(B,D,E)**, the values plotted are the mean value and standard deviation from triplicate experiments.

The unique cell wall structure of *Mycobacterium tuberculosis* (Mtb) is the basis for adhesion, recognition and defense, and it also plays an important role for survival (Daffé and Draper, 1998). Three kinds of macromolecules, including mycolic acids, arabinogalactan (AG), and peptidoglycan (PG), compose the mycobacterial cell wall core (McNeil and Brennan, 1991; Brennan, 2003). As an important glycosyl donor, UDP-N-acetylglucosamine (UDP-GlcNAc) is involved in the attachment of AG and PG through a disaccharide linker (a-L-rhamnosyl-a-D-N-acetylglucosaminosyl-1-phosphate), and it is also the precursor for the synthesis of PG (Li et al., 2012). Four-step reactions are required in the biosynthesis of UDP-GlcNAc, and the last two sequential biosynthetic steps are catalyzed by the bifunctional enzyme glucosamine-1-phosphate acetyltransferase/N-acetylglucosamine-1-phosphate uridyltransferase (GlmU). GlmU acetyltransferase activity converts GlcN-1-P and Acetyl CoA into GlcNAc-1-P and CoA, then GlmU uridyltransferase activity catalyzes the synthesis of UDP-GlcNAc from GlcNAc-1-P and UTP (Figure 1A; Mengin-Lecreulx and Van Heijenoort, 1993, 1996; Kang et al., 2013). The essentiality of *glmU* in Mtb had been demonstrated by the transposon site hybridization (TraSH) method (Sassetti et al., 2003), and it was knocked out in *Mycobacterium smegmatis* where it led to growth inhibition and morphological changes of bacterial cells (Zhang et al., 2008). Therefore, GlmU is a potential anti-TB drug target.

Recent publications reported some compounds that inhibit the uridyltransferase or acetyltransferase activities of Mtb GlmU. Tran et al. reported that active aminoquinazoline-based compounds targeted the uridyltransferase activity of Mtb GlmU. The most potent inhibitor exhibited an IC_50_ of 74 μM (Tran et al., 2013). Further, Soni and co-workers used the isothermal titration calorimetry (ITC) method to find that the binding energy pattern of compound 4 (IC_50_: 42.07 μM) to the uridyltransferase active site of Mtb GlmU was similar to that of substrate UTP (Soni et al., 2015a). Soni et al. also found a novel Mtb GlmU uridyltransferase inhibitor Oxa33 (IC_50_: 9.96 μM), and Oxa33 inhibited the *in vitro* growth of Mtb H37Rv with a MIC of 75 μM (Soni et al., 2015b). However, the uridyltransferase activity of Mtb GlmU catalyzed the same reaction as UDP-N-GlcNAc pyrophosphorylase AGX1 and AGX2 in humans (Peneff et al., 2001). Although the homology between Mtb GlmU and AGX1 and AGX2 was low, the drug development of uridyltransferase activity of Mtb GlmU should be cautious. Since the reaction catalyzed by Mtb GlmU acetyltransferase activity is absent in humans, we consider that Mtb GlmU acetyltransferase is a more suitable target for anti-TB drug development.

Mehra et al. (2016) applied structure and ligand-based computational models to screen a druglike compound repository of 20,000 compounds for the identification of probable inhibitors of Mtb GlmU. The IC_50_ of the most potent identified inhibitory lead (5810599) was found to be 9.018 μM (Mehra et al., 2016). Rani et al. found four compounds which showed an inhibitory effect on GlmU acetyltransferase activity, with IC_50_ values ranging from 9 to 70 μM. Compounds 6624116 and 5655606 exhibited activity against drug-susceptible as well as drug-resistant Mtb (Rani et al., 2015). However, the detailed information of the action mechanism of these compounds was not reported.

From our screening results, we found that compound TPSA showed an inhibitory effect on the acetyltransferase activity of Mtb GlmU. Here, we attempted to investigate its action mechanism on Mtb GlmU acetyltransferase and its inhibitory effect on Mtb H37Ra as well as the proteome and transcriptome changes of Mtb H37Ra treated with TPSA. Since we found that the expression of two methyltransferases encoded by MRA_0565 (Rv0558 in H37Rv, demethylmenaquinone methyltransferase) and MRA_0567 (Rv0560c in H37Rv, benzoquinone methyltransferase) was increased greatly, we also investigated the possible methylation of TPSA by methyltransferases.

## Materials and Methods

### Bacterial Strains and Plasmids

*Mycobacterium tuberculosis* H37Ra strain (ATCC 25177) and *M. smegmatis* mc^2^155 strain (ATCC 700084) were obtained from American type culture collection. *Escherichia coli* NovaBlue (Novagen) was used for gene cloning and BL21(DE3) (Novagen) was for GlmU protein expression. Cloning plasmid pJET1.2 blunt (Thermo) with the ampicillin resistance gene was used for cloning genes and re-sequencing the target genes. Expression vectors pET16b (Novagen) carrying the kanamycin resistance gene were utilized to express the GlmU protein in *E. coli* BL21(DE3) and pColdII (Takara) carrying ampicillin resistance gene was utilized to express Rv0558 and Rv0560c proteins in BL21(DE3). Mycobacterial expression vector pVV2 was used to express GlmU, Rv0558 and Rv0560c protein in H37Ra. Mycobacterial expression vector pMind was used to downregulate GlmU in *Mycobacterium smegmatis*. *M. tuberculosis* H37Rv genomic DNA was supplied by Colorado State University via an NIH contract.

### Materials

Mtb strains were grown in Difco Middlebrook 7H9 (BD Biosciences) medium with 10% (vol/vol) ADC (0.5% BSA; Roche, 0.2% dextrose, 0.0003% catalase), 0.8% glycerol, and 0.005% polysorbate 80, or plated on 7H11 agar (BD Biosciences) supplemented with 10% (vol/vol) ADC (0.5% BSA; Roche, 0.2% dextrose, 0.0003% catalase) and 1% glycerol. Compounds 2-[5-(2-{[4-(2-thienyl)-2-pyrimidinyl]sulfanyl}acetyl)-2-thienyl]acetic acid (TPSA), 4-chloro-N-[2-chloro-4-({4′-[(3-chloro-4-{[(4-chlorophenyl)sulfonyl]amino}phenyl) sulfonyl][1,1′-biphenyl]-4-yl} sulfonyl) phenyl] benzenesulfonamide (CBBS), 1-[3-(2,5-dioxo-2,5-dihydro-1H-pyrrol-1-yl)phenyl]-2,5-dihydro-1H-pyrrole-2,5-dione (DHPP), 6,12-dihydrobenzo[b]chromeno[4,3-e][1,4]thiazin-6-one (BCTZ), 5-{4-[(4-nitrobenzyl)oxy]benzylidene}-2-thioxo-1,3-thiazolan-4-one (NBTT), 4-nitroindane-1,3-dione (NID) and 3-(2,5-dimethyl-1H-pyrrol-1-yl)thiophene-2-carboxylic acid (MPTC) were purchased from Maybridge company (Cornwall, United Kingdom). The Ni-NTA superflow column used for protein purification was from QAIGEN. The MutanBEST Kit used for *glmU* mutation was obtained from Takara Biotechnology (Dalian). The (anti)-polyhistidine clone HIS-1 antibody, the substrates AcCoA, GlcN-1-P and S-adenosylmethionine (SAM) for enzyme assay were purchased from Sigma-Aldrich Chemical and the Goat anti-mouse IgG (H+L)-AP conjugate was obtained from Proteintech.

### Preparation of GlmU Protein

*Escherichia coli* BL21(DE3) carrying pET16b-*glmU* plasmid was used to express GlmU protein, BL21(DE3)/ pET16b-*glmU* was cultured in LB broth with 50 μg/ml ampicillin and induced with 1 mM IPTG at 30°C for 6 h (Zhang et al., 2008). The cells were harvested and suspended in lysis buffer (20 mM Tris-HCl, pH 8.0, 500 mM NaCl, 20% glycerol, 1 mM EDTA, and 1 mM PMSF) followed by sonication. After centrifugation at 20,000×g for 15 min, the supernatant was applied to Ni-NTA superflow column (Qiagen). The column was then washed with 20 ml wash buffer (lysis buffer with 45 mM imidazole) and GlmU protein with a His-tag at its C-terminus was eluted by 10 ml elution buffer (lysis buffer with 200 mM imidazole). The concentration of GlmU protein was determined by the Bradford method, using BSA as standard. The purified GlmU protein was detected by SDS-PAGE and Western Blotting. GlmU protein was run on the 12% SDS-PAGE and transferred to a nitrocellulose membrane in transfer buffer (20 mM Tris-base, 150 mM glycine and 20% methanol). The membrane was incubated with blocking buffer (10 mM Tris-HCl, pH 8.0, 150 mM NaCl, 0.05% Tween 20 and 10% Nonfat dry milk) and then incubated with (anti)-polyhistidine clone HIS-1 antibody at 1:5000 dilution. The membrane was incubated with antimouse-IgG-AP conjugate at a dilution of 1:3000, and the GlmU protein bands were visualized in BCIP/NBT solution.

### Inhibition of GlmU Acetyltransferase Activity by TPSA

The acetyltransferase activity of GlmU protein was detected by DTNB [5, 5′-dithiobis-(2-nitrobenzoic acid)] colorimetric assay as previously described (Zhou et al., 2011). The reaction mixture containing 0.04 μg purified GlmU protein, 5 mM MgCl_2_, 50 mM Tris-HCl (pH 7.5), 0.4 mM glucosamine-1-phosphate (GlcN-1-P), 0.4 mM acetyl coenzyme A (AcCoA) and 20% glycerol was incubated at 37°C for 5 min. Then the reaction was stopped with a stop solution containing 50 mM Tris-HCl (pH 7.5) and 6 M guanidine hydrochloride. After adding a colorimetric reagent solution containing 0.2 mM DTNB, the absorbance values at wavelength of 405 nm were obtained using a microplate reader. The control containing all components except GlmU protein was used to correct the errors from the -SH group of GlmU, and a positive control containing 0.2 mM CoA was used for calculating the amount of CoA produced in reactions. Using DTNB colorimetric assay, GlmU acetyltransferase inhibitors were screened from 5000 compounds provided by Maybridge and J&K Scientific as well as 2000 natural products provided by Shanghai Aladdin Bio-Chem Technology and prepared by College of Pharmacy at Dalian Medical University. Briefly, compounds were dissolved in DMSO solvent and added to reaction mixture at a final concentration of 100 μM for preliminary screening. GlmU was pre-incubated with different compounds on ice for 5 minutes and then GlmU activity was detected. The enzyme reaction only containing DMSO solvent was used as a negative control. All experiments were performed in triplicate.

### Inhibition Types of TPSA

To investigate the inhibition types, the dual-substrate reactions of GlmU with TPSA at IC_50_ and two-folds of IC_50_ were performed. The inhibitory constant (*Ki*) and inhibition types were determined by double reciprocal plots. Molecular docking was also used to predict the interaction type of GlmU and TPSA.

### Interaction of GlmU and TPSA by Molecular Docking and Site-Directed Mutagenesis

The 3D structure of GlmU (PDB code: 4G3S) was obtained from PDB database (https://www.rcsb.org/). The interaction sites of GlmU and TPSA complexes were visualized by LIGPLOT program and PyMOL (version 1.3) (Wallace et al., 1995). AutoDockTools (ADT) was used to transform the pdb format receptor into the pdbqt format and the Mol2 format ligand which could be used in AutoDock (version 4.2). The receptor docking gridbox was also set using the ADT, whose number grid points in XYZ were 126 × 126 × 126, and spacing was 0.375Å. The Lamarckian Genetic Algorithm (LGA) of AutoDock was selected as the docking simulated method, and 100 runs were performed in this study (Morris et al., 2009).

Site-directed mutagenesis experiments were then performed to verify the docking results. Ten amino acid residues (Phe340, Tyr342, Phe358, Val359, Leu375, Thr376, Tyr377, Ser392, Ser393, and Val394) of GlmU were predicted to be important for binding with TPSA. Therefore, these amino acid residues were substituted to alanine using the MutanBEST Kit, respectively. All GlmU mutant proteins were expressed, purified, quantified and their enzyme activity was determined by method mentioned above.

### MIC Determination

To test whether TPSA had an inhibitory effect on the growth of Mtb H37Ra, Mtb clinical isolates and *Mycobacterium smegmatis*, microdilution alamarBlue assay (MABA) was used as described previously (Dkhar et al., 2015). The alamarBlue dye is able to indicate the cellular growth and metabolism of any bacteria based on the color conversion from blue to pink. Briefly, the 50 μl 7H9 broth containing compounds at final concentrations ranging from 6.25 to 100 μg/ml were added in the 96-well microtiter plates. The bacterial culture suspension (2 × 10^6^ cfu/ml) was prepared by mixing bacterial stock with 7H9 broth and 50 μl of culture suspension was then added into the above wells. The culture only containing DMSO was used as control. After the culture was incubated for 7 days at 37°C, 100 μl of 0.01% alamarBlue solution was added to each well and the color of culture was observed after an additional 3–5 h of incubation. If the alamarBlue color remained blue, bacterial growth was considered to be inhibited. The lowest concentration of the compounds at which bacterial growth was inhibited was considered as the minimum inhibitory concentration (MIC). The MIC detection of Mtb clinical isolated strain was completed in the National Clinical Laboratory on Tuberculosis, Beijing Key Laboratory on Drug-Resistant Tuberculosis Research.

To further investigate whether GlmU was targeted by TPSA, *glmU* was cloned to a mycobacterial expression vector pVV2 to generate pVV2-*glmU*, then pVV2- *glmU* was electroporated into Mtb H37Ra, resulting in H37Ra/ pVV2-*glmU*. The empty vector pVV2 was also electroporated into H37Ra as control. MABA was then used to monitor the growth of H37Ra/pVV2-*glmU* and H37Ra/pVV2 when treated with different concentrations of TPSA.

pMind, mycobacterial express vector can be used to express a gene or antisense RNA in mycobacteria under the induction of tetracycline (Blokpoel et al., 2005). In our previous studies, we established a downregulated *glmU* expression strain (Msm/pMind-As *glmU*) by using *Mycobacterium smegmatics* (Msm), which is a non-pathogenic and fast-growing mycobacterium. Msm and Mtb have similar structures of cell wall, therefore, Msm as a model has been used to identify gene function, test biofilm formation, investigate the effect of antimicrobial susceptibilities, etc. (Zhang et al., 2008; Kang et al., 2013). The Msm *glmU* gene (MSMEG_5426) was found to have 75% identical homology with the Mtb *glmU* gene (*Rv1018c*) using BLASTP (Zhang et al., 2008). We used Msm/pMind-As *glmU* to examine whether TPSA targeted GlmU in Msm. The method was followed as previously described. Msm/pMind-As *glmU* was grown in LB broth with 0.05% Tween 80 and kanamycin at 37°C. Msm GlmU expression was downregulated by adding 10 ng/ml tetracycline. The bacteria culture without tetracycline was used as the control. The culture was plated on a 96-well plate and TPSA at different concentrations was added. After 24 h incubation at 37°C, the MIC of TPSA to Msm/pMind-As *glmU* was observed by the MABA method.

### Bacterial Integrity Testing

The effect of the inhibitor TPSA on bacterial integrity was assessed using propidium iodide (PI) staining (Sharma et al., 2016). PI is the most regularly used as a fluorescence indicator for living prokaryotic or eukaryotic cell viability based on the membrane permeability (Yang et al., 2015). H37Ra was grown overnight at 37°C and when the cells were grown to an OD (600 nm) of 0.5, TPSA was treated for 24 h at a concentration of 25 μg/ml. Samples were centrifuged (3,000 rpm, 4°C, 10 min) and the pellets were washed twice with 1 ml saline solution. The pellets were then resuspended in 1 ml saline solution, stained with 3 μl of 20 mM propidium iodide dye mixture (Sigma) and incubated for 15 min in dark. Then, the PI dye was removed by centrifugation, the bacterial cells were washed with 1 ml saline solution, and 100 μl of the cells was observed under fluorescence microscope (OLYMPUS) by using a 10 × lens.

### Scanning Electron Microscopy and Transmission Electron Microscopy

Scanning electron microscopy (SEM) analysis of untreated Mtb H37Ra and TPSA treated Mtb H37Ra were performed as described earlier (Zhang et al., 2008). SEM images were captured using JSM-6369-LV scanning electron microscope. Transmission electron microscopy (TEM) was performed using standard protocols. Briefly, untreated Mtb H37Ra and TPSA treated Mtb H37Ra bacteria were fixed in 2.5% glutaraldehyde, dehydrated in graded series of alcohol and then embedded in the epoxy resin. Ultrathin sections were cut and stained using uranyl acetate and lead citrate. Finally, TEM images were procured using a JEM-1400 PLUS transmission electron microscope.

### Drug Combination Testing

MABA was also used to test the effect of combination between TPSA and the three anti-TB first line drugs isoniazid (INH), rifampicin (RFP), ethambutol (EMB). The effect of drug combinations was categorized based on a fractional inhibitory concentration (FIC) of ≤ 0.5 as synergistic effect, a of > 0.5 and ≤ 1 as additive, a of > 1 and < 4 as no drug combination effect, and a of ≥ 4 as antagonistic action (Hall et al., 1983; Sukheja et al., 2017). Moreover, 10 μl of cultures was taken for determining the CFU between TPSA and three first-line drugs isoniazid, rifampicin and ethambutol.

### Cytotoxicity Testing

The 3-[4, 5-dimethylthiazol-2yl]-2, 5-dipheny tetrazolium bromide (MTT) assay and TransDetect^TM^ Cell Counting Kit (CCK) assay were utilized to test the cytotoxicity of TPSA on RAW264.7 and THP-1 macrophage cells separately. RAW264.7 cells were grown in the wells of a 96-well plate (10^5^ cells/well) containing DMEM medium plus 10% fetal bovine serum (FBS), while THP-1 cells were grown with RPMI 1640 medium plus 10% FBS. The medium containing different concentrations of TPSA was then added to the culture followed by incubation for 2 days. MTT was added in each well for an additional 4 h of incubation, and OD_595_ was read by a microplate reader. Meanwhile, CCK was added in the wells and OD_450_ was recorded. The medium containing DMSO was used as control. The CC_50_ value (concentration causing 50% cell cytotoxicity) and SI value (the ratio between CC_50_ and the MIC data was calculated which explains the safety index of an inhibitor as an estimate of the therapeutic window) of TPSA were calculated (Sharma et al., 2016).

### Infection of H37Ra to Macrophages Cells

pCG76-*GFP* vector (constructed in our lab) was electroporated into H37Ra, generating H37Ra/pCG76-*GFP*. The macrophage cells RAW264.7 were seeded at 1 × 10^5^ cells per well in a 24-well plate. After incubation for 12 h, macrophage cells were infected with H37Ra/pCG76-*GFP* at a multiplicity of infection (MOI) of 1:10 for 4 h. Then, the intracellular H37Ra/pCG76-*GFP* was treated with TPSA (1 × MIC and 2 × MIC) for 24 h after being washed three times with PBS. The cells were observed and photographed by fluorescence microscope after DAPI (DNA-binding dye) staining. Then the number of cells which contained H37Ra/pCG76-*GFP* were counted. Cells used for bacterial counting were lysed in PBS containing 0.05% SDS for 10 min. The lysates were plated on 7H11 agar plates and the CFU results were counted after 4 weeks.

### Two-Dimensional Electrophoresis (2-DE)

The 2-DE was performed in triplicate for both TPSA treated samples and DMSO control samples to minimize artifacts and errors (Rao et al., 2014; Yari et al., 2017). Log-phase culture of H37Ra was treated with 25 μg/ml of TPSA or with DMSO for 24 h. The protein was extracted and performed as described above and a 2-D Clean-up Kit (GE Healthcare) was used to remove interfering substances such as detergents, salts, lipids, phenolics, and nucleic acids from the protein samples. Then, the protein concentration was measured by using Coomassie Brilliant Blue staining. For the first dimension, 40 μg of protein samples were mixed with rehydration solution (8 M urea, 2% CHAPS, 0.5% IPG buffer and 0.002% bromophenol blue). The resulting solution was then used to rehydrate 7 cm IPG strips (linear pH 4-7; GE Healthcare) for 12 h. Isoelectric focusing was carried out using the IPG phore plate (BioRad) at room temperature for a total of 28375 Vh (250 V for 30 min, 500 V for 30 min, 500–4,000 V for 2 h, 4,000 V for 20,000 Vh). IPG strips were equilibrated for 30 min with equilibration buffer (6 M urea, 75 mM Tris-HCl (pH 8.8), 29.3% glycerol (v/v) and 0.002% bromophenol blue). For the second dimension, two strips were transferred to 12% SDS-PAGE and gels were initially run at 50 V for 1 h and then at 130 V until bromophenol blue reached the bottom of the gel. The gels were then fixed in the fixing solution (50% absolute ethyl alcohol and 10% acetic acid) for 2.5 h and stained using ProteoSilver^TM^ Plus Silver Stain Kit (Sigma). Significant spots were found and excised from all six gels. These protein spots were then excised manually and subjected to protein digestion and peptide extraction, followed by MALDI-/ MS analysis.

### RNA Extraction and qRT-PCR Analysis

Log-phase culture of H37Ra was treated with 25 μg/ml of TPSA or with negative control (DMSO) for 24 h. Total RNA was extracted from H37Ra using RNAisoplus (Takara). Briefly, the aqueous layer was transferred and isopropanol was added to precipitate the RNA, and the resulting pellet was washed twice with 75% ethanol. RNA was dissolved in the DEPC-treated water and the RNA quantity was detected using NanoDrop (Thermo). Quantitative real-time PCR (qRT-PCR) was then used to validate the differential expression of genes identified by 2-DE results. The first-strand cDNA was synthesized using the *TransScript* One-Step gDNA Removal and cDNA Synthesis SuperMix (TRANSGEN BIOTECH) with 1 μg of total RNA. The diluted cDNA was used as a template for qRT-PCR, using the *TransStart* Top Green qPCR SuperMix (TRANSGEN BIOTECH) supplemented with designed primers of different genes. PCR amplification was performed for quantification of gene expression levels. Primer sequences were listed in Supplementary Table 2. The fold change in the expression of genes was calculated by the 2^−ΔΔ*CT*^ method and the expression of *Rv2703c* (*SigA*), a house-keeping gene, was used as the normalization control (Wang et al., 2017).

### RNA-Seq Analysis

Total RNA was extracted from H37Ra using Trizol reagent (Life Technologies) method and RNA was quantified by the ND-1000 NanoDrop (Thermo Scientific). rRNA was removed from the total RNA using the EpicentreRibo-Zero rRNA Removal Kit (Illumina), according to manufacturer's instructions. After fragmentation and random priming, first and second strand cDNA was synthesized and purified according to the instructions of NEBNext® Ultra^TM^ RNA Library Prep Kit for Illumina (New England Biolabs). After adapter ligation and PCR amplification, the quality test of the library was performed by an Agilent 2200 TapeStation (Agilent Technologies). Finally, the RNA sequencing was performed with a Hiseq 2500 sequencer (Illumina). The sequenced reads were aligned to the complete genome of H37Ra (National Center for Biotechnology Information database, NCBI). The transcript abundances were measured in reads per kilobase of exon per million mapped reads (RPKM). Upregulated or downregulated genes were identified based on the criteria that expression levels differed by over two-folds relative to control and RPKM>10 in all samples.

### Cloning and Expression of Rv0558 and Rv0560c Protein

*MRA_0565* in H37Ra was 100% identical to *Rv0558* in H37Rv and *MRA_0567* in H37Ra was 100% identical to *Rv0560c* in H37Rv. *Rv0558* and *Rv0560c* were amplified from Mtb H37Rv genomic DNA and cloned into pJET1.2 blunt vector, yielding pJET-*Rv0558* and pJET-*Rv0560c* plasmids for resequencing. Expression vectors pColdII-*Rv0558* and pColdII-*Rv0560c* were constructed for expression of Rv0558 or Rv0560c protein in *E. coli*. Rv0558 and Rv0560c proteins were purified by Ni-NTA superflow column (Qiagen). After quantification by Bradford method, proteins were detected by SDS-PAGE and Western Blotting. *Rv0558* and *Rv0560c* were cloned to expression vector pVV2 to generate pVV2-*Rv0558* and pVV2-*Rv0560c*, respectively. pVV2- *Rv0558* and pVV2-*Rv0560c* were electroporated into Mtb H37Ra, resulting in H37Ra/ pVV2-*Rv0558* and H37Ra/pVV2-*Rv0560c*.

### Methylation of TPSA by Rv0558 and Rv0560c

TPSA with different concentrations was pre-incubated with 0.6 μM Rv0558 or Rv0560c in the presence of 100 μM SAM on ice for 5 min and then GlmU acetyltransferase reaction was performed. The enzyme reaction only containing DMSO solvent was used as a negative control, and the reaction without Rv0558 and Rv0560c was as positive control. All experiments were performed in triplicate.

After methylation reaction at 37°C for 1 h, the mixture was added to the H37Ra culture and then the growth of H37Ra was monitored by MABA. The susceptibility of H37Ra/pVV2-*Rv0558* and H37Ra/pVV2-*Rv0560c* to TPSA was also tested.

To detect whether TPSA was methylated by Rv0558 or Rv0560c, methylation reaction was incubated at 37°C for 14 h. The methylated product was detected using liquid chromatography-mass spectrometry (LC-MS) (AB Sciex 5500 Q-trap). Chromatographic column was balanced with mobile phase (50% acetonitrile + 0.1% formic acid). Reaction solutions were separated at a flow rate of 200 μl/minute. The samples were separated using HPLC eluted at gradient mobile phase (50–100% acetonitrile, 10 min). The mass spectrometer data were analyzed using ESI in the positive ion mode with a selected mass range of 100–1,000 (*m/z*), and the survey scans were acquired at a resolution of 60,000. Normalized collision energy was 1.5 KV with a collision mode of CID. The instrument was set at data-dependent automatic collecting mode.

### Statistical Analysis

Statistical analysis between groups was performed using unpaired two-tailed *t*-test by GraphPad Prism v.6.01 software. All measurements were presented as the mean ± SD obtained from three independent experiments to correct the trial errors.

## Results

### Inhibition Type of TPSA on GlmU Acetyltransferase Activity Was Determined

The DTNB colorimetric assay was used to screen GlmU acetyltransferase inhibitors. Seven compounds (TPSA, CBBS, DHPP, BCTZ, NBTT, NID, MPTC) showed inhibitory activity against GlmU acetyltransferase and TPSA exhibited a lower inhibition concentration on GlmU acetyltransferase compared to the other six compounds. The IC_50_ (5.3 μM) of TPSA against GlmU was observed (Figure 1B). The structural formula of TPSA was shown in Figure 1C. The inhibitory constant (*Ki*) and inhibition types of TPSA on GlmU were determined by double reciprocal plots (Table 1 and Figures 1D,E). Kinetic studies of TPSA on GlmU showed that the inhibition type was mixed with GlcN-1-P and competitive with AcCoA. The structural formula of the other six compounds and their IC_50_ value, inhibitory constant (*Ki*) and inhibition types on GlmU acetyltransferase are shown in Supplementary Figure 1, Supplementary Table 1.

**Table 1:** The inhibition of TPSA on GlmU acetyltransferase.

	**IC_**50**_ (μM)**	***K***_***i***_ **(μM)**			
		***K_***i*−*com***_*/ GlcN-1-P**	***K_***i*−*non***_*/ GlcN-1-P**	***K_***i*−*com***_*/ AcCoA**	***K_***i*−*non***_*/ AcCoA**
TPSA	5.30 ± 0.48	30.46 ± 0.0013	2.46 ± 0.0038	8.27 ± 0.0014	–

### Interactions Between GlmU and TPSA Were Analyzed

**Figure 2:**
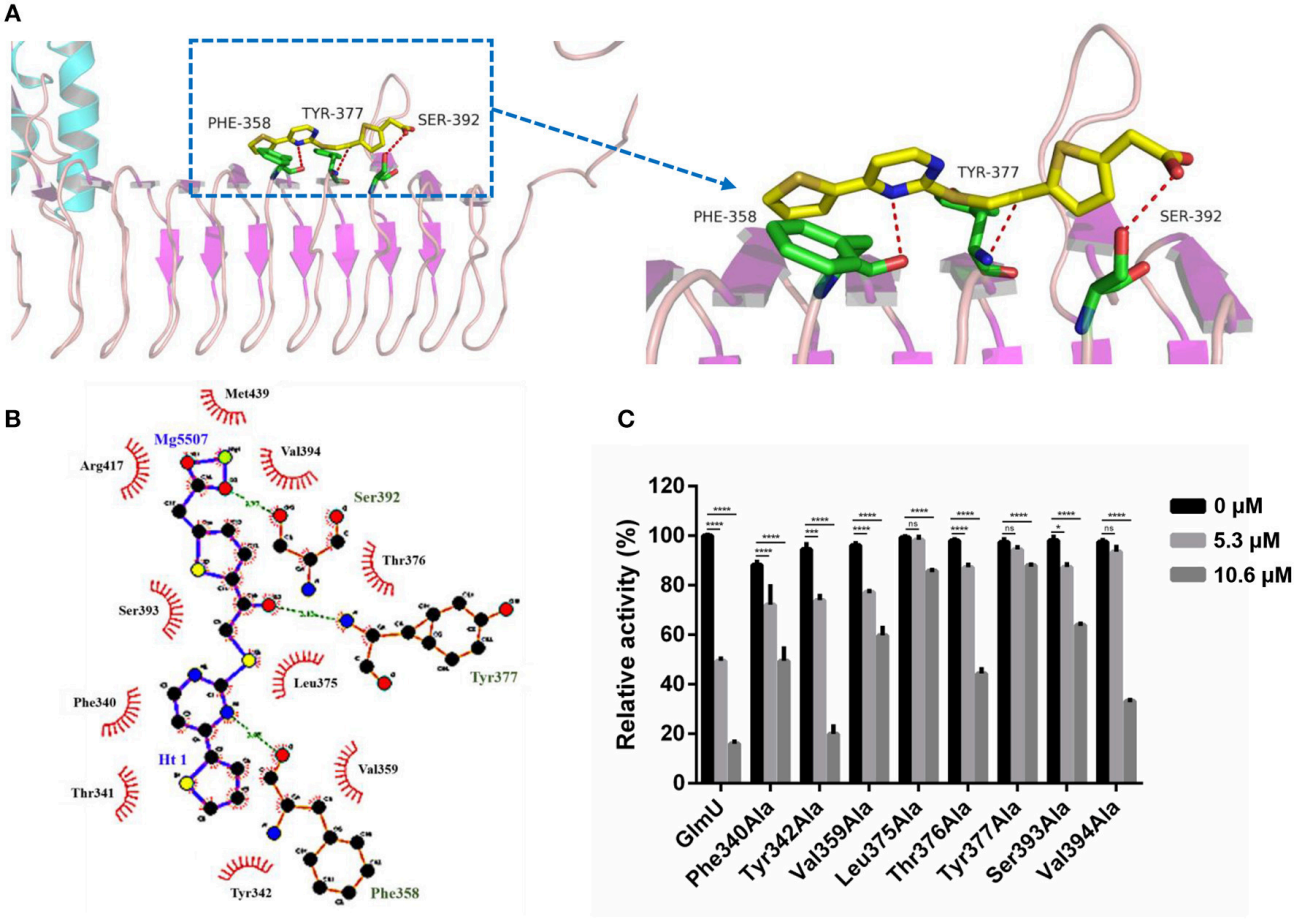
Post-docking interactions between active site residues of GlmU acetyltransferase with TPSA **(A,B)** and the relative activity of different GlmU protein mutants **(C)**. In **(C)**, the experiment was carried out in triplicate and the graph indicates the mean value with standard deviation bars. Differences among groups were calculated by unpaired two-tailed *t*-test. The asterisks represent the statistical differences between the relative activity of GlmU and GlmU mutants in different concentrations of TPSA-treated group. ns, no significance; *P < 0.05; ***P < 0.001; ****P < 0.0001 (The *P-*values of untreated group to 5.3 μM TPSA treated group for GlmU, *P* < 0.0001; Phe340Ala, *P* < 0.0001; Tyr342Ala, *P* < 0.0001; Val359Ala, *P* < 0.0001; Leu375Ala, *P* = 0.5572; Thr376Ala, *P* < 0.0001; Tyr377Ala, *P* = 0.0815; Ser393Ala, *P* = 0.0012 and Val394Ala, *P* = 0.3158. The *P-*values of untreated group to 10.6 μM TPSA treated group for GlmU, *P* < 0.0001; Phe340Ala, *P* < 0.0001; Tyr342Ala, *P* < 0.0001; Val359Ala, *P* < 0.0001; Leu375Ala, *P* < 0.0001; Thr376Ala, *P* < 0.0001; Tyr377Ala, *P* < 0.0001; Ser393Ala, *P* < 0.0001 and Val394Ala, *P* < 0.0001).

The docking simulation results revealed the interactions between TPSA and amino acid residues of GlmU acetyltransferase. Three amino acid residues (Phe358, Tyr377, and Ser392) formed hydrogen bonds with TPSA (Figure 2A). Two-dimensional representation of these interactions, plotted by LIGPLOT, is shown in Figure 2B. The results from LIGPLOT indicated that TPSA was predicted to form stable hydrogen bonds with the amino group of Phe358, Tyr377, and Ser392. In addition, hydrophobic bonding interactions of seven amino acid residues (Phe340, Tyr342, Val359, Leu375, Thr376, Ser393, and Val394) were found at the perimeter of TPSA. According to molecular docking analysis of GlmU and TPSA binding sites, ten residues of GlmU, which were shown to be bound with TPSA, were substituted by alanine. GlmU mutant proteins Phe340Ala, Tyr342Ala, Phe358Ala, Val359Ala, Leu375Ala, Thr376Ala, Tyr377Ala, Ser392Ala, Ser393Ala, and Val394Ala were expressed and purified (Supplementary Figures 2A,B). Unfortunately, soluble Ser392Ala failed to be expressed. The activities of mutant GlmU acetyltransferase were detected using the above-mentioned method. Mutant proteins Phe358Ala completely lost the GlmU acetyltransferase activity, indicating Phe358 is important for the catalytic activity of GlmU acetyltransferase (Supplementary Figure 2C). When these mutant GlmU proteins were incubated with different concentrations of TPSA, the inhibitory effect of TPSA was decreased compared with the wild type GlmU (Figure 2C). These results provided further evidence for the critical role of these eight residues for binding of GlmU with TPSA.

### TPSA Had Inhibitory Effect on H37Ra Growth

**Figure 3:**
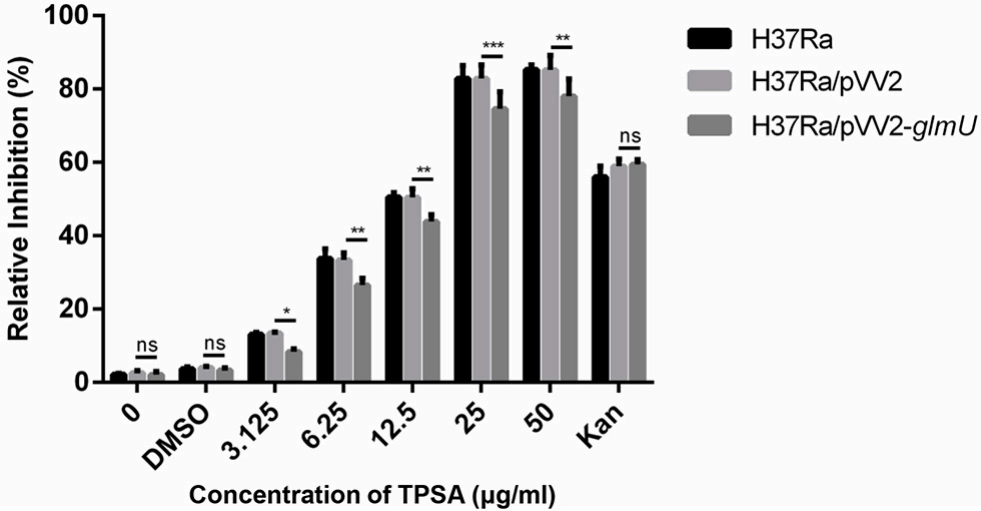
Growth inhibition of H37Ra/ pVV2-*glmU* treated with different concentration TPSA for 24 h. The experiment was performed in triplicates and error bars indicate standard deviation. Differences among groups were calculated by unpaired two-tailed *t*-test. The asterisks represented the statistical differences between the relative inhibition of H37Ra/pVV2 and H37Ra/pVV2-*glmU* in different concentrations of the TPSA-treated group. ns, no significance; *P < 0.05; **P < 0.01; ***P < 0.001 (0, *P* = 0.8339; DMSO, *P* = 0.7273; 3.125 μg/ml, *P* = 0.0156; 6.25 μg/ml, *P* = 0.0018; 12.5 μg/ml, *P* = 0.0024; 25 μg/ml, *P* = 0.0002; 50 μg/ml, *P* = 0.001; Kan, *P* = 0.8169).

TPSA was subsequently tested for its efficacy against H37Ra, Mtb clinical isolates, and *M. smegmatis* using MABA. TPSA had a MIC of 25 μg/ml against H37Ra, 25 μg/ml against Mtb clinical isolated strain 3326, 50 μg/ml against Mtb clinical strain 5148 and 50 μg/ml against *M. smegmatis*, suggesting a potential anti-TB compound. To further investigate whether GlmU was targeted by TPSA, pVV2*-glmU* plasmid was constructed to overexpress GlmU in H37Ra. Compared to the H37Ra containing only an empty vector, the inhibitory effect of TPSA on H37Ra/pVV2-*glmU* was decreased slightly (Figure 3). Kanamycin was used a control to indicate that the inhibitory effect of kanamycin on H37Ra/pVV2 and H37Ra/pVV2-glmU was same. The results suggested that TPSA could target GlmU in H37Ra.

In addition, Msm/pMind-As *glmU* was used to examine whether TPSA targeted GlmU in Msm. The map of Msm/pMind-As *glmU* is shown in Supplementary Figure 3A. The MIC of TPSA to Msm/pMind-As *glmU* without tetracycline was 50 μg/ml whereas it was 12.5 μg/ml for Msm/pMind-As *glmU* with tetracycline. The inhibitory effect of TPSA on *glmU* down-regulated expression strain was increased, which suggested that TPSA targeted GlmU in Msm (Supplementary Figure 3B).

### Bacterial Wall and Membrane Integrity Was Destroyed by TPSA

**Figure 4:**
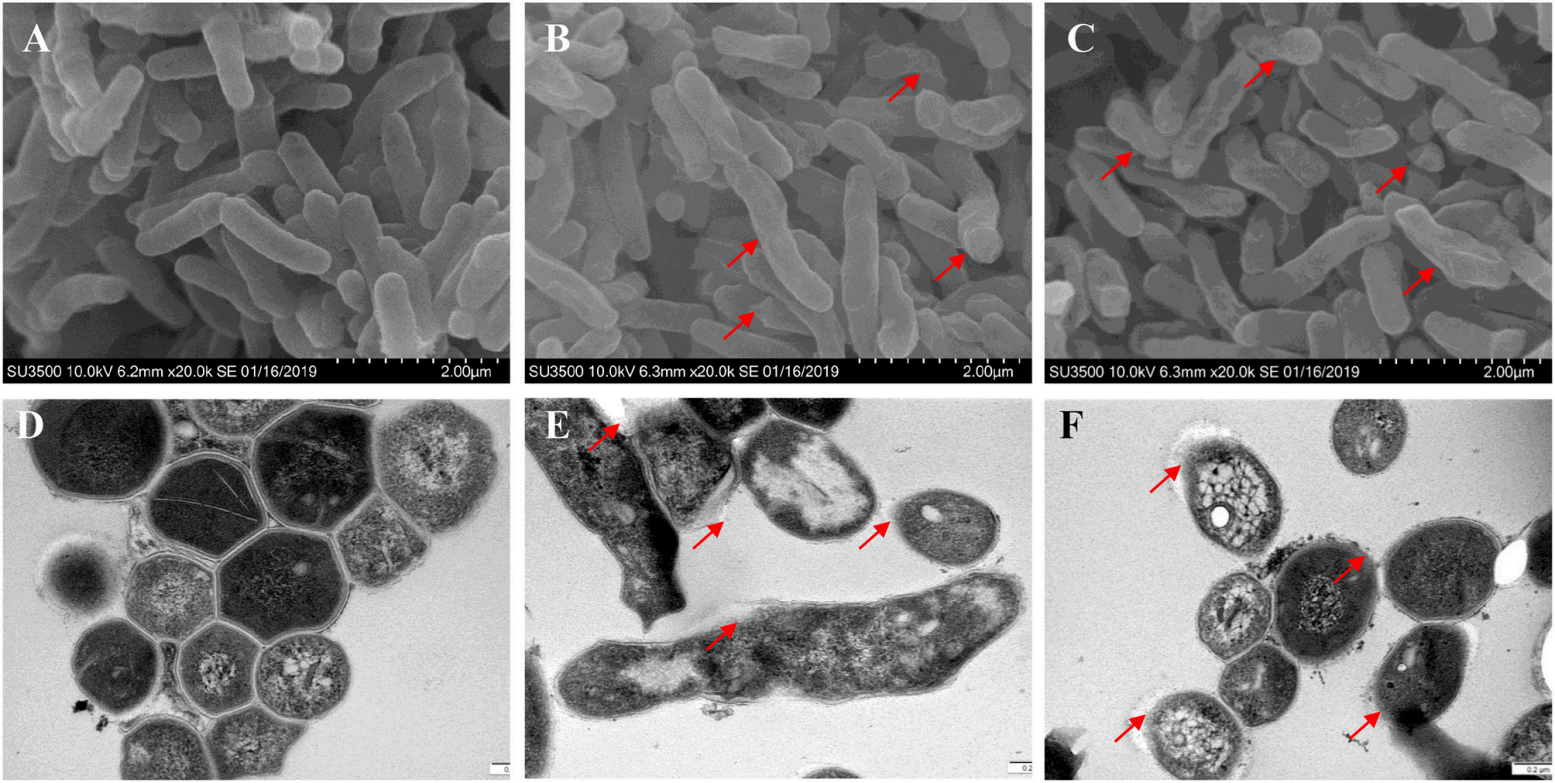
Scanning electron microscopy and transmission electron micrographs of Mtb H37Ra cells. SEM of untreated cells of Mtb H37Ra **(A)** and Mtb H37Ra treated with TPSA (2×MIC) for 24 h **(B,C)** (20,000×). Red arrows in **(B,C)** depict the unsmooth and wrinkled surface of bacilli. TEM of untreated cells of Mtb H37Ra **(D)** and Mtb H37Ra treated with TPSA (2×MIC) for 24 h **(E,F)** (80,000×). Red arrows in **(E,F)** point to the areas of bacilli lysis or cell wall rupture.

Bacterial membrane integrity is essential for its survival, and therefore it is frequently used as a viability indicator. To determine whether TPSA had influence on the cell wall and membrane of H37Ra, PI staining was used to bind with DNA and can be observed with red fluorescence (Supplementary Figure 4). Bacterial cells treated with TPSA at 2 × MIC (50 μg/ml) concentration showed uptake of PI indicating that TPSA could permeate the cell wall and cell membrane of H37Ra and destroy their integrity, whereas untreated cells showed little PI uptake. To further determine the impact of TPSA on cellular morphology and cell wall structure, the SEM and TEM images of untreated Mtb H37Ra cells and 2 × MIC TPSA treated Mtb H37Ra cells were analyzed (Figure 4). SEM analysis revealed morphological change in the TPSA-treated group, with the bacilli showing a wrinkled surface and fused cells (Figures 4B,C). TEM analysis showed that untreated and TPSA-treated Mtb H37Ra cell wall structure and thickness are comparable, and there was a marked decrease in cell wall thickness and bacilli lysis in the TPSA-treated group (Figures 4E,F). These SEM and TEM results suggest that TPSA was able to affect the morphology and cell wall structure of Mtb H37Ra.

### TPSA Had a Synergistic Effect With the First-Line TB Drug Rifampin

**Figure 5:**
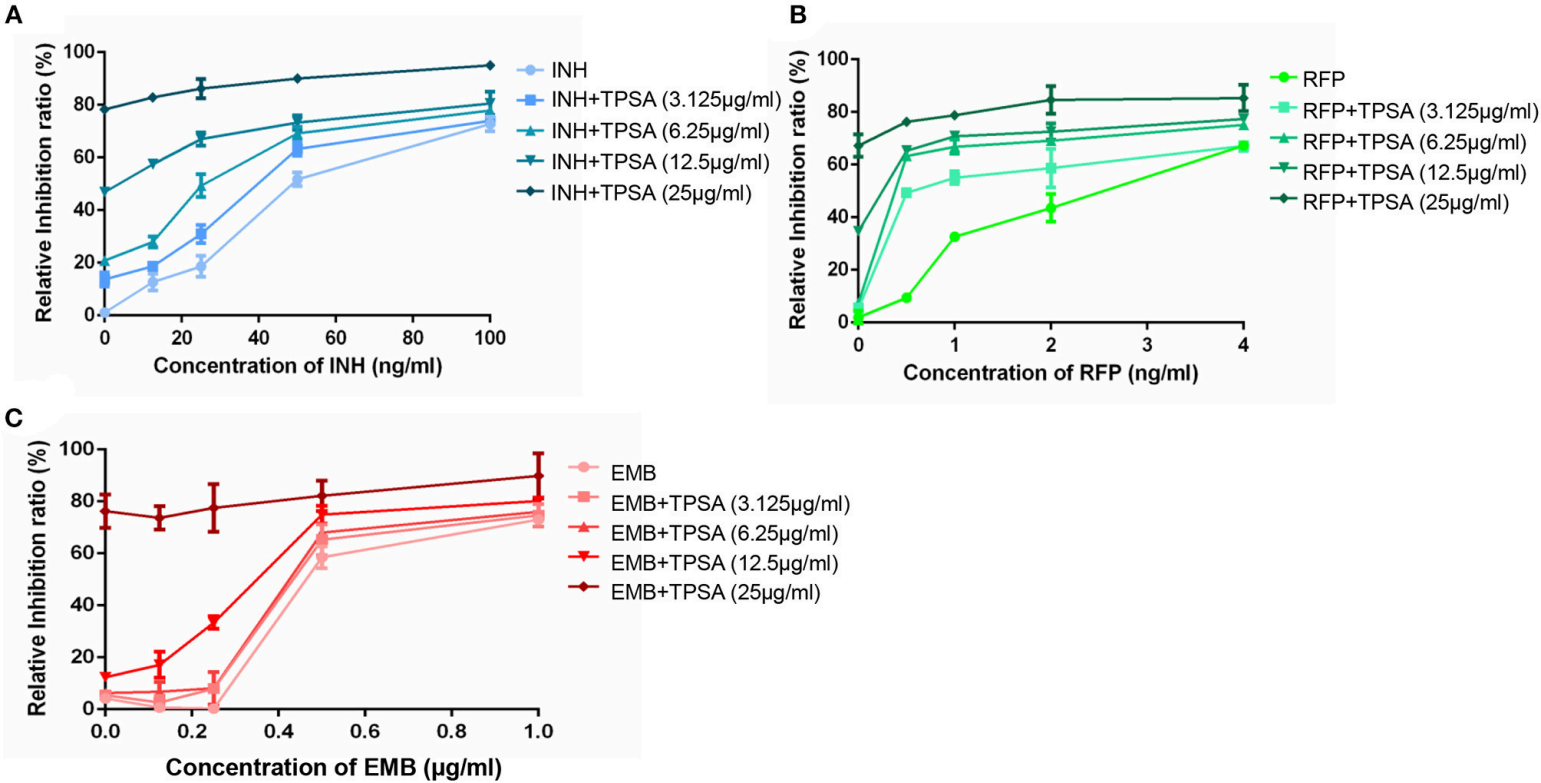
The relative inhibition ratio of the synergistic effect on H37Ra between TPSA and three first-line drugs isoniazid **(A)**, rifampicin **(B)**, and ethambutol **(C)**. Data are representative of one experiment with three independent biological replicates (mean and SD).

The synergistic effect of TPSA with three first-line drugs (INH, RFP and EMB) was examined by the MABA method. The relative inhibition ratios and the *in vitro* bactericidal activity of the synergistic effect are shown in Figure 5, Supplementary Figure 5, and Table 2. From the results, we observed that the MIC of the drug combination group was decreased compared to that of each drug alone. Synergistic effect was shown in the group treated by RFP and TPSA with FIC (fractional inhibitory concentrations) of 0.35. The interactions of TPSA with INH and EMB were additive, with FIC values of 0.75 and 0.98, respectively.

**Table 2:** Synergy between TPSA and anti-TB first line drugs.

**Drug combination**	**MIC (μg/ml)**		FIC^*^	**FIC index**
	**Alone**	**In combination**		
INH	0.048	0.025	0.52	0.75
TPSA	25	6.34	0.25	Additive
RFP	0.0023	0.0051	0.22	0.35
TPSA	25	3.173	0.13	Synergistic
EMB	0.46	0.37	0.8	0.98
TPSA	25	4.6	0.18	Additive

* *Drug combinations were categorized based on a fractional inhibitory concentration (FIC) of ≤ 0.5 as synergistic effect, a FIC of > 0.5 and ≤ 1 as additivity, a FIC of > 1 and < 4 as drug no interaction, and a FIC of ≥ 4 as antagonistic action*.

### TPSA Had Weak Cytotoxicity on THP-1 and RAW264.7 Macrophage Cells

Cytotoxicity assay was performed on human macrophage cell THP-1 and mouse macrophage cell RAW264.7 to evaluate whether TPSA had toxicity to mammalian macrophage cells. Supplementary Figure 6 did not show significant cytotoxic effects (CC_50_ > 150 μg/ml). TPSA exhibited cytotoxicity with CC_50_ of 303.54 ± 1.86 μg/ml and 178.91 ± 1.57 μg/ml to THP-1 and RAW264.7 macrophage cells. In addition, TPSA showed an acceptable SI of 12.12 and 7.20 to THP-1 and RAW264.7 macrophage cells, respectively.

### TPSA Was Able to Inhibit Growth of Intracellular H37Ra in RAW264.7 Macrophage

**Figure 6:**
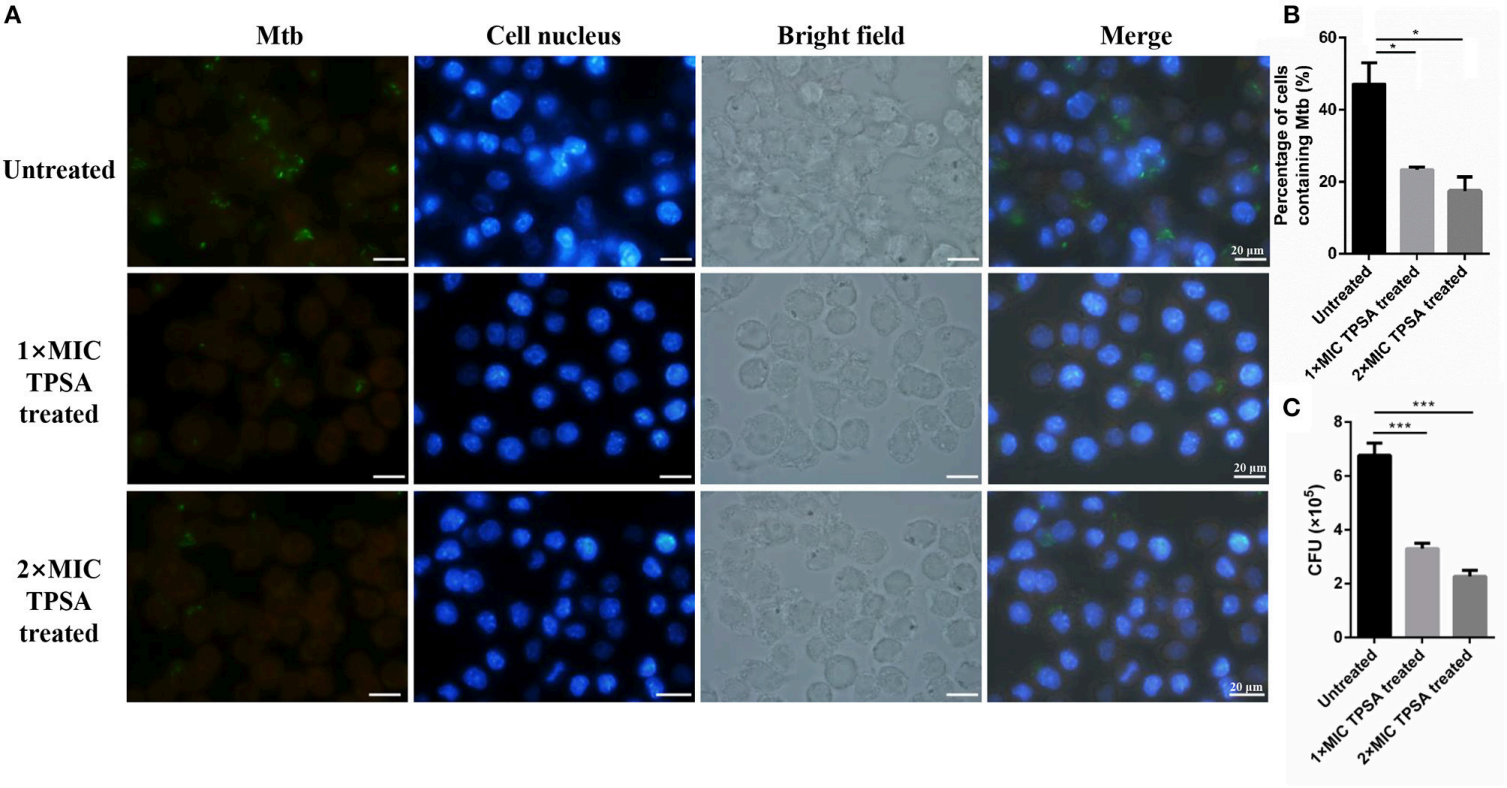
Fluorescence microscope of RAW264.7 cells infected with H37Ra/pCG76-*GFP* strains **(A)**. Cells were infected at multiplicity of infection (MOI) of 1:10 for 4 h. H37Ra in macrophage was then treated with TPSA (1 × MIC and 2 × MIC) for 24 h. The green color of H37Ra/pCG76-*GFP* could be detected. Cell nucleus was stained with DNA-binding dye (DAPI) (blue). The percentage of cells containing H37Ra (a total of 252 cells were counted) **(B)** and the survival of H37Ra and H37Ra/pCG76-*GFP* strains in RAW264.7 cells **(C)**. In **(B,C)**, the experiment was performed in triplicate, and error bars indicate standard deviation. Differences among the groups were calculated by unpaired two-tailed *t*-test. The asterisks represented the statistical differences between the percentage of cells containing H37Ra or the quantity of H37Ra in the different concentrations of the TPSA-treated group. *P < 0.05; ***P < 0.001 [In **(B)**, the *P* value of the untreated group to 1 × MIC TPSA treated group is 0.02 and the *P-*value of the untreated group to 2 × MIC TPSA treated group is 0.0102. In **(C)**, the *P-*value of the untreated group to 1 × MIC TPSA treated group is 0.0003 and the *P-*value of untreated group to 2 × MIC TPSA treated group is 0.0001].

TPSA also inhibited intracellular H37Ra growth in RAW264.7 macrophage cells at different concentrations (Figure 6A). The green fluorescence of H37Ra/pCG76-*GFP* and blue fluorescence of cell nuclei stained with DAPI (DNA-binding dye) were observed. The percentage of cells containing H37Ra (a total of 252 cells in per group was counted) and CFU results were presented in Figures 6B,C.

### H37Ra Proteome Profile Changed in Response to TPSA Treatment

2-DE and MALDI-/ MS were used to investigate the proteome profile of TPSA-treated H37Ra and untreated H37Ra. Proteins were separated using isoelectric-focusing electrophoresis in the first dimension and SDS-PAGE in the second dimension. After 2-DE, the gels were stained with silver staining kit and protein pattern was analyzed by using the ImageMaster 2D Platinum 5 software. A total of 274 differentially expressed and statistically significant protein spots were identified (*P* < 0.05). Among these spots, 258 spots exhibited down-regulation (ranging from 19.46 folds to 2.02 folds) and 16 showed upregulation (ranging between 1.52 folds to 10.20 folds) in samples which had received TPSA treatment. Seven protein spots that were significantly differentially expressed in the treatment group were selected (Supplementary Figures 7A,B). These spots were then digested and subjected to mass spectrometry for identification and characterization. The results are presented in Table 3, three of the identified proteins, spot no. 215 and 216 — 3-oxoacyl-(Acyl-carrier-protein) synthase 1 (KasA, −4.55 and −2.02 folds), spot no. 206 — Acyl-CoA dehydrogenase (FadE25, −2.70 folds) and spot no. 203 — 3-ketoacyl-(Acyl-carrier-protein) reductase (FabG-1, −4.93 folds) showed downregulated expression (spot no. 215 and 216 were identified as the same protein). KasA catalyzed the condensation reaction of fatty acid synthesis by the addition to an acyl acceptor of two carbons from malonly-. FadE25 was involved in the first cycle of side chain dehydrogenation in the beta-oxidation of cholesterol catabolism. FabG-1 took part in the fatty acid elongation. In addition, three proteins were upregulated, spot no. 224—enolase (MRA_1031, 1.79 folds), spot no. 197−3-ketoacyl-(Acyl-carrier-protein) reductase (FabG-1, 1.91 folds) and spot no. 491—uncharacterized protein (MRA_0567, 10.20 folds). Enolase catalyzed the reversible conversion of 2-phosphoglycerate into phosphoenolpyruvate, and it was essential for the degradation of carbohydrates via glycolysis. MRA_0567 (Rv0560c in H37Rv) had the benzoquinone methyltransferase activity, and it could be induced by salicylate and at higher concentrations by para-aminosalicylate (Denkin et al., 2005; Gamngoen et al., 2018).

**Table 3:** List of significant differentially expressed proteins identified in TPSA-treated H37Ra using 2-D electrophoresis and MALDI/TOF-TOF.

**Spot ID**	**Gene name**	**Uniprot ID**	**Protein name**	**MW** **(kDa)**	**Protein PI**	***P*-value** **(*t*-test)**	**No. of matched peptides**	**Protein score**	**Total ion score**	**Flod change**
224	*MRA_1031*	A5U166	Enolase	44.93	4.50	0.017	16	551	471	1.79
215	*MRA_2265* (*kasA*)	A5U4S7	3-oxoacyl-(Acyl-carrier-protein) synthase 1	43.29	5.11	0.003	13	303	243	−4.55
216	*MRA_2265* (*kasA*)	A5U4S7	3-oxoacyl-(Acyl-carrier-protein) synthase 1	43.29	5.11	0.015	10	486	442	−2.02
206	*MRA_3315* (*fadE25*)	A5U7U8	Acyl-CoA dehydrogenase	41.70	5.21	0.019	16	132	35	−2.70
203	*MRA_0251* (*fabG-1*)	A5TYW8	3-ketoacyl-(Acyl-carrier-protein) reductase	46.83	6.04	<0.0001	13	432	389	−4.93
197	*MRA_0251* (*fabG-1*)	A5TYW8	3-ketoacyl-(Acyl-carrier-protein) reductase	46.83	6.04	0.014	15	366	294	1.91
491	*MRA_0567*	A5TZU0	Benzoquinone methyltransferase	25.93	4.68	<0.0001	14	807	713	10.20

### qRT-PCR Validation

The differentially expressed proteins identified from 2-DE results were verified at the mRNA expression level. All the primers designed for qRT-PCR are presented in Supplementary Table 2. The relative fold changes were compared to the untreated H37Ra group, and the results are presented in Supplementary Figure 7C. All these five genes exhibited upregulated expression in H37Ra treated by 50 μg/ml TPSA for 24 hours. *MRA_1031, kasA, fadE25*, and *fabG-1* were found to be upregulated by 2.86-fold, 1.16-fold, 3.17-fold, and 2.28-fold as detected by qRT-PCR, respectively. Unpredictably, *MRA_0567* presented the largest increase in expression (843.69-fold). The qRT-PCR results thus drew our attention to the upstream or downstream genes of *MRA_0567*, including *MRA_0564, MRA_0565, MRA_0566*, and *MRA_0568*. Three of these genes, *MRA_0565* (61.82-fold), *MRA_0566* (112.99-fold), and *MRA_0568* (5.58-fold) were also upregulated (Supplementary Figure 7D).

### H37Ra Transcriptome Profile Changed in Response to TPSA Treatment

To investigate the changes of the H37Ra transcriptome profile in response to TPSA treatment, RNA-seq was performed. Differentially expressed genes were filtered using the false discovery rate (FDR) of < 0.05 and a log_2_ fold change > 1 or < −1 for biological significance. After filtering, three genes were found to be significantly upregulated, and eight genes were downregulated (*P* < 0.0001) when treated with TPSA (Table 4 and Supplementary Figure 8A). Regarding the functional categories, most of these genes were involved in the biological processes and molecular functions (Supplementary Figure 8B). Three genes with increased expression were *MRA_0565, MRA_0566*, and *MRA_0567*. These results were consistent with the proteome results. Meanwhile, the genes with diminished expression were *MRA_RS02635, MRA_0731, MRA_1749, MRA_2046, rnpB, Novel 00016* (a novel transcript) and *sRNA00033*. MRA_0565 (Rv0558 in H37Rv, MenG, demethylmenaquinone methyltransferase) is responsible for the conversion of demethylmenaquinone (DMK9) to menaquinone (MK9) and required for respiration (Berney et al., 2015).

**Table 4:** List of differentially expressed genes identified in TPSA-treated H37Ra using RNA-Seq.

**Gene id**	**Readcounts_TPSA**	**Readcounts_Untreated**	**Description**	***P-*value**	**Flod change**
*MRA_0565*	932.223550776929	76.9968072220299	Demethylmenaquinone methyltransferase	<0.0001	12.11
*MRA_0566*	1743.06733236726	170.115689602463	Hypothetical protein	<0.0001	10.25
*MRA_0567*	5670.52178636248	100.346019520407	Benzoquinone methyltransferase	<0.0001	56.51
*MRA_RS02635*	51.6032290442103	124.251165444936	AURKAIP1/COX24 domain-containing protein	<0.0001	−2.41
*MRA_0731*	151.780801949601	306.041461196588	50S ribosomal protein L15	<0.0001	−2.02
*MRA_1749*	1451.28472640206	2966.73979595929	Hypothetical protein	<0.0001	2.04
*MRA_2046*	733.214576158605	1518.25473302068	Alpha-crystallin	<0.0001	−2.07
*MRA_2366*	17.9489492327688	56.4272630544118	ESAT-6 like protein EsxO	<0.0001	−3.14
*rnpB*	5405.55042331373	13216.2100945077	RNase P RNA component class A	<0.0001	−2.44
*Novel 00016*	408.5629569109	842.517410433114		<0.0001	−2.06
*sRNA00033*	107.244971665794	215.702246946914		<0.0001	−2.01

### Rv0558 and Rv0560c Affected the Inhibitory Effect of TPSA

**Figure 7:**
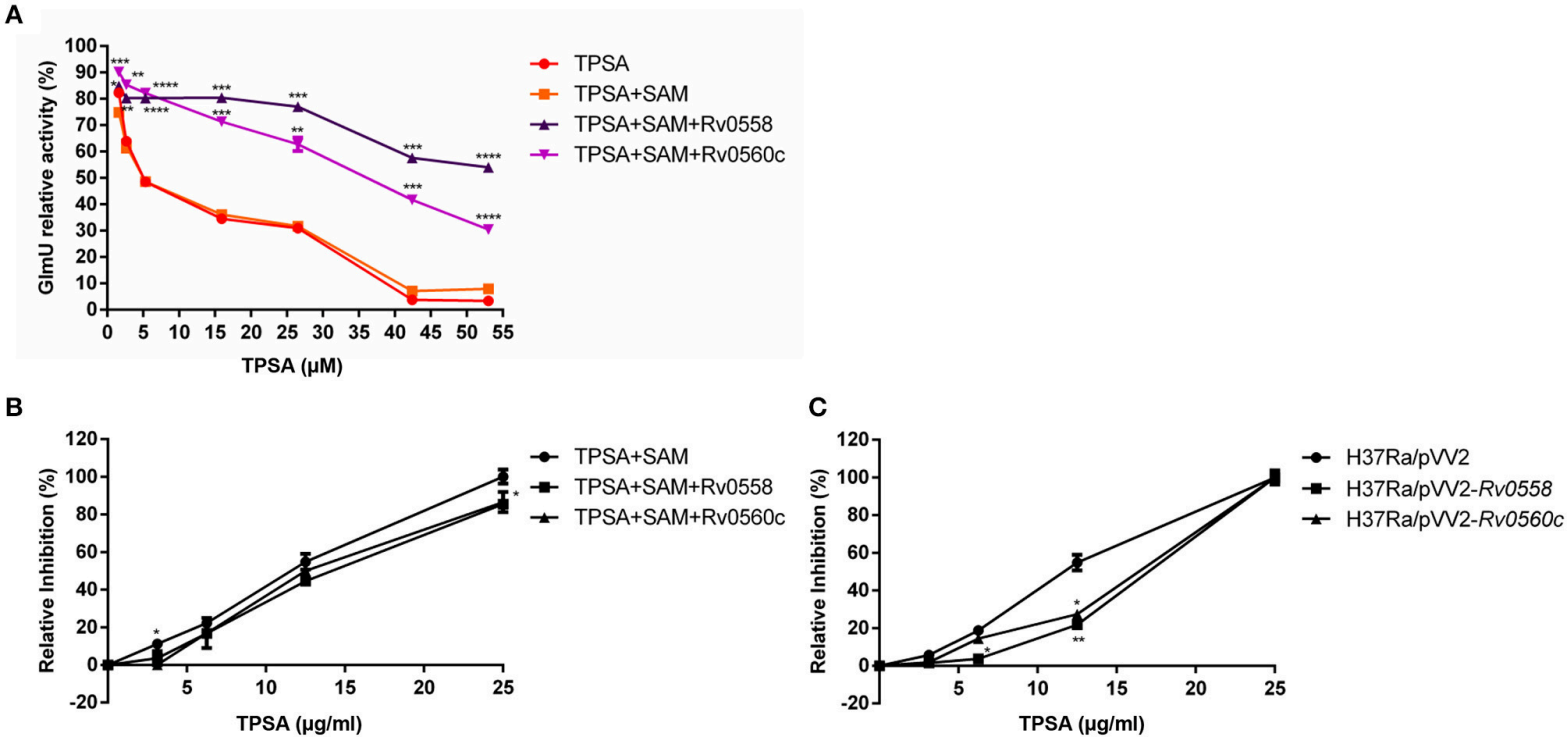
Detection of GlmU relative activity **(A)** and relative inhibition of H37Ra growth **(B)** when treated with TPSA and SAM-dependent methylation of TPSA by Rv0558 protein and Rv0560c protein. Detection of relative growth inhibition of H37Ra/pVV2-*Rv0558* and H37Ra/pVV2-*Rv0560c* strain as well as H37Ra/pVV2 strain as a control when treated with different concentrations of TPSA **(C)**. Differences among the groups were calculated by unpaired two-tailed *t*-test. *P < 0.05; **P < 0.01; ***P < 0.001; ****P < 0.0001 [In **(A)**, the *P-*values of TPSA + SAM group to TPSA + SAM + Rv0558 group for 1.59 μM, *P* = 0.0174; 2.65 μM, *P* = 0.0072; 5.3 μM, *P* < 0.0001; 15.9 μM, *P* = 0.0001; 26.5 μM, *P* = 0.0002; 42.4 μM, *P* = 0.0001 and 53 μM, *P* < 0.0001. The *P-*values of TPSA + SAM group to TPSA + SAM + Rv0560c group for 1.59 μM, *P* = 0.0006; 2.65 μM, *P* = 0.0068; 5.3 μM, *P* < 0.0001; 15.9 μM, *P* = 0.0001; 26.5 μM, *P* = 0.0037; 42.4 μM, *P* = 0.0006 and 53 μM, *P* < 0.0001. In **(B)**, the *P-*values of TPSA + SAM group to TPSA + SAM + Rv0558 group for 0, *P* > 0.9999; 3.125 μg/ml, *P* = 0.1178; 6.25 μg/ml, *P* = 0.0776; 12.5 μg/ml, *P* = 0.0793 and 25 μg/ml, *P* = 0.035. The *P* values of TPSA + SAM group to TPSA + SAM + Rv0560c group for 0, *P* > 0.9999; 3.125 μg/ml, *P* = 0.0203; 6.25 μg/ml, *P* = 0.4582; 12.5 μg/ml, *P* = 0.2466 and 25 μg/ml, *P* = 0.0999. In **(C)**, the *P-*values of H37Ra/pVV2 group to H37Ra/pVV2-*Rv0558* group for 0, *P* = 0.9964; 3.125 μg/ml, *P* = 0.1284; 6.25 μg/ml, *P* = 0.016; 12.5 μg/ml, *P* = 0.0082 and 25 μg/ml, *P* = 0.9994. The *P-*values of H37Ra/pVV2 group to H37Ra/pVV2-*Rv0560c* group for 0, *P* = 0.9985; 3.125 μg/ml, *P* = 0.1041; 6.25 μg/ml, *P* = 0.1532; 12.5 μg/ml, *P* = 0.0113 and 25 μg/ml, *P* = 0.9995].

Based on the putative methyltransferase activity of Rv0558 and Rv0560c, a hypothesis was proposed in our study. TPSA inhibited the acetyltransferase activity of GlmU; however, when TPSA came into the bacteria, two methyltransferases Rv0558 and Rv0560c could methylate it, resulting in reduced inhibitory effect of TPSA. To verify the hypothesis, the two methyltransferases were expressed in *E. coli* and purified (data not shown). If TPSA was methylated by purified Rv0558 or Rv0560c protein, then the inhibition of methylated TPSA on GlmU acetyltransferase activity could be changed. The decreased inhibitory effect of TPSA incubated with Rv0558 and Rv0560c protein was observed from the GlmU relative activity shown in Figure 7A.

The results also showed that H37Ra became less sensitive to TPSA at 25 μg/ml (MIC) after incubation of Rv0558 or Rv0560c (Figure 7B). H37Ra/pVV2-*Rv0558* and H37Ra/pVV2-*Rv0560c* strains overexpressed Rv0558 and Rv0560c protein, respectively. TPSA showed weaker inhibitory effect on H37Ra/pVV2-*Rv0558* and H37Ra/pVV2-*Rv0560c* compared to H37Ra/pVV2 at the 12.5μg/ml (0.5 × MIC) (Figure 7C).

**Figure 8:**
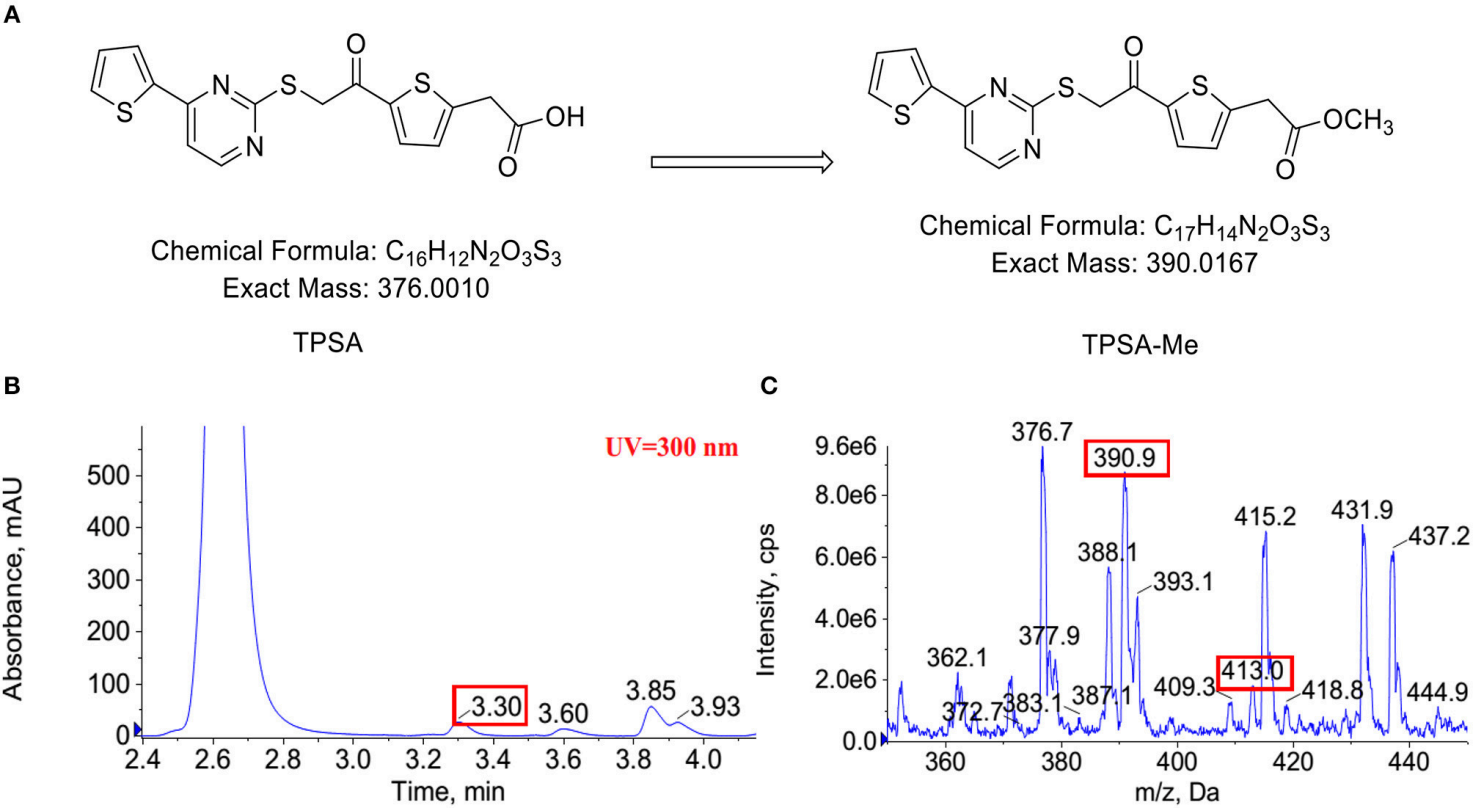
The predicted methylation of TPSA **(A)**. Methylation of TPSA confirmed by LC-MS after 14 h co-incubation of TPSA and Rv0560c in the presence of SAM. The methylated product had a retention time of 3.3 min at HPLC-UV (300 nm) **(B)**. The mass spectrum of the methylated product corresponding to 3.3 min: (+) ion peak *m/z* 390.9 [M + H]^+^ and *m/z* 413.0 [M + Na]^+^ **(C)**.

Indeed, an additional peak with *m/z* of 390 in the reaction of Rv0560c was detected by LC-MS. The product was 14 mass units greater than TPSA, which indicated the hydroxyl group of TPSA was substituted by a methyl group (Figure 8). Unfortunately, no obvious peak for methylated TPSA in the reaction of Rv0558 was detected (data not shown). The results suggested that Rv0560c was able to modify TPSA by methylation. Therefore, TPSA needs to be modified by some groups to prevent its methylation by methyltransferases. Five derivatives of TPSA were designed to avoid methylation (Supplementary Figure 9). In future work, we will evaluate the inhibitory effect of TPSA derivatives on GlmU acetyltransferase and Mtb.

## Discussion

The rigid cell wall of bacteria protects them from a variety of environmental and host threats (Soni et al., 2015b). The enzymes involved in the biosynthetic pathway of bacterial cell wall are considered attractive targets for anti-microbial therapies because of their essentiality, and most of them do not have any homologous protein in mammals (Lovering et al., 2012).

UDP-GlcNAc is an essential precursor of PG and the rhamnose-GlcNAc linker region in the mycobacterial cell wall. Bifunctional enzyme GlmU is a bifunctional enzyme responsible for the last two steps of UDP-GlcNAc biosynthetic pathway. GlmU acetyltransferase converts GlcN-1-P to GlcNAc-1-P and subsequently GlmU uridyltransferase converts the GlcNAc-1-P to UDP-GlcNAc. The essentiality of *glmU* in mycobacteria had been demonstrated by Transposon site hybridization (TraSH) methodology and knockout experiment (Sassetti et al., 2003; Zhang et al., 2008). Therefore, GlmU can be a potential target for the development of new anti-tuberculosis drugs.

The high-throughput screening (HTS) method of Mtb GlmU acetyltransferase activity (DTNB assay) and Mtb GlmU uridyltransferase activity (pyrophosphatase-coupled malachite green assay) was established in our previous studies (Zhou et al., 2011). Mammals do not have the same reaction catalyzed by GlmU acetyltransferase activity. Thus, we have been focused on inhibitors for Mtb GlmU acetyltransferase in our laboratory and found several compounds by using DTNB assay. TPSA exhibited inhibitory activity against GlmU acetyltransferase with an IC_50_ of 5.3 μM. Kinetic studies of TPSA on GlmU acetyltransferase showed that the inhibition was competitive with substrate AcCoA and mixed with GlcN-1-P. The inhibitory effect of TPSA on GlmU uridyltransferase was also checked in this study. As expected, TPSA showed no inhibitory effect on GlmU uridyltransferase (data not shown).

According to the Lipinski's Rule of Five (a rule of thumb to assess whether a compound can be used as a drug or a compound with pharmacological or biological activity as an oral drug), the general properties of TPSA as a lead are as follows. The molecular weight of TPSA is 376 daltons and the H bond donors and acceptors of TPSA are 1 and 4, respectively. The lipid-water partition coefficient of TPSA is 5 and the number of rotatable bonds is 8. TPSA exhibited weak cytotoxicity on THP-1 and RAW264.7 macrophage cells. Additionally, molecular docking and site-directed mutagenesis revealed that three amino acid residues (Phe358, Tyr377, and Ser392) of GlmU acetyltransferase formed hydrogen bonds with TPSA. These results suggested that TPSA as a GlmU acetyltransferase inhibitor may offer a structural basis for developing new anti-tuberculosis drugs. We also found that mutant protein Phe358Ala completely lost the GlmU acetyltransferase activity, indicating that Phe358 was a key active site of GlmU acetyltransferase and TPSA was able to compete with substrate to inhibit the acetyltransferase activity of GlmU. These results were helpful for us to understand the inhibitory mechanism of TPSA.

TPSA was capable to inhibit Mtb H37Ra and Mtb clinical isolates even though its MIC was higher the IC_50_. TPSA acted on GlmU target in Mtb H37Ra carrying pVV2-glmU. Therefore, TPSA destroyed the integrity of the Mtb H37R cell wall through interfering the formation of UDP-GlcNAc, a donor for linker and precusor for peptidoglycan. Interestingly, TPSA had synergistic effect with RFP and were additive with INH and EMB, respectively, though the action site of RFP was RNA polymerase, but both INH and EMB acted on mycobacterial cell wall.

From the data of proteomics, qRT-PCR and transcriptome analyses, we were interested in two putative methyltransferases, MRA_0565 and MRA_0567, and they were 100% identical to Rv0558 and Rv0560c in H37Rv, respectively. Rv0558 catalyzed the final step of menaquinone biosynthesis and it was identified as a high-confidence drug target. Menaquinone and its saturated form [MK-9 (II-H2)] were the only lipoquinones in Mtb that transfer electrons from the dehydrogenases to terminal electron oxidases. Paridhi Sukheja et al. found that GSK1733953A (DG70) is an inhibitor of Rv0558. DG70 had an MIC of 4.8 μg/ml against Mtb H37Rv and 1.2 to 9.6 μg/ml against drug-resistant strains (Upadhyay et al., 2015; Sukheja et al., 2017). Rv0560c was a putative benzoquinone methyltransferase, and it can be induced by salicylate (Sukheja et al., 2017; Gamngoen et al., 2018). Rv0560c was reported to methylate pyrido-benzimidazole “14,” which had inhibitory effect against DprE1 that was involved in Mtb arabinogalactan synthesis (Warrier et al., 2016). Rachel Kokoczka et al. reported that Rv0560c was not essential for growth (Kokoczka et al., 2017), and it implied that Rv0560c was not a good drug target, but the high expression of Rv0560c may play an important function under a chemical stress condition.

We supposed that when TPSA came into the bacteria, two methyltransferases Rv0558 and Rv0560c methylated it, therefore reducing its inhibitory effect on bacterial growth. Both enzyme assay of GlmU acetyltransferase and growth of H37Ra/pVV2-*Rv0558* and H37Ra/pVV2-*Rv0560c* gave us evidence that TPSA inhibition was attenuated due to action of Rv0558 or Rv0560c on TPSA. However, the methylation of TPSA only by Rv0560c was confirmed by LC-MS. It may be that the amount of methylated TPSA was too small to detect the peak in the Rv0558 group. The results suggested that TPSA was methylated by Rv0560c with methyltransferase activity.

In conclusion, an inhibitor TPSA of GlmU acetyltransferase was found, and it also had an inhibitory effect on H37Ra growth and low cytotoxicity to mammalian cells. However, since inhibitory function of methylated TPSA on Mtb was reduced, it needs to be modified to avoid the methylation catalyzed by methyltransferases, e.g. Rv0560c in the further studies. Of course, the structure-activity relationship of modified TPSA also needs to be investigated. It is also necessary to find the inhibitors of methyltransferase Rv0560c. The combination of TPSA and Rv0560c inhibitors may show better inhibitory effect against Mtb.

## Data Availability

All datasets generated for this study are included in the manuscript and the Supplementary Files.

## Author Contributions

All authors of this manuscript have seen and approved the content, and have contributed significantly to this work. YM, CC, and XH contributed to the conception, design and writing. CC, XH, QY, LJ, AT, and LZ performed the experiments. CC, XH, QY, and CW conducted data analysis and prepared the figures.

### Conflict of Interest Statement

The authors declare that the research was conducted in the absence of any commercial or financial relationships that could be construed as a potential conflict of interest.
