# Supplementary material for: The Inhibitory Effect of GlmU Acetyltransferase Inhibitor TPSA on Mycobacterium tuberculosis May Be Affected Due to Its Methylation by Methyltransferase Rv0560c
Source: Front Cell Infect Microbiol. 2019 Jul 17;9:251. doi: 10.3389/fcimb.2019.00251 (PMC6652808; doi:10.3389/fcimb.2019.00251)
Supplement: Supplementary file 1 [file Table_1.docx]

Supplementary table 1. The inhibition results of different compounds on GlmU acetyltransferase

|  | IC_50_ (µM) | *K_i_* (µM) | | | |
| --- | --- | --- | --- | --- | --- |
|  |  | *K_i-un_* / GlcN-1-P | *K_i-non_* / GlcN-1-P | *K_i-com_* / AcCoA | *K_i-non_* / AcCoA |
| CBBS | 5.88 | 4.5 | ─ | 5.5 | ─ |
| DHPP | 10.28 | ─ | 26.27 | 26.43 | ─ |
| BCTZ | 61.67 | ─ | 82.83 | ─ | 81.56 |
| NBTT | 65.10 | ─ | 41.27 | 40.18 | ─ |
| NID | 124.13 | 19.72 | ─ | ─ | 80.86 |
| MPTC | 158.52 | ─ | 41.08 | 139.53 | 26.49 |

Supplementary table 2. The sequences of primers

| Primers | Forward primers | Reverse primers |
| --- | --- | --- |
| *MRA_1031* | CACTGTCCGAAGACGATTGG | GCTCGGGATTGGTGACAAAG |
| *kasA* | CCGACCCTGAACTACGAGAC | CCCGAACGAGTTGTTGACTG |
| *fadE25* | GCGGCGGAGTAAACCATCAG | CAGTTCGGTGAGTCGATCAG |
| *fabG-1* | TGTTGGCCAGCAGCTTGTCAC | ACGATGCCGTCGACAAGATCAG |
| *MRA_0567* | TGTCGAACCTGCCGTCATAG | AGAACTGGCTCGGCATGAAG |
| *MRA_0564* | AGCTACGGCATTCCGATGAC | CCCTGGGCAATAAGGGATTC |
| *MRA_0565* | ATGCGGTCACCATCAGTTTC | GAATTCGCACACTAGTAGCC |
| *MRA_0566* | TCCATCGCTTCGGGATCTACG | GGAAAGTGGCCGACTTGTACG |
| *MRA_0568* | CTGACCTTCCCACGGTTCCTAC | GTCGTCGTCGGTGACCAGATTGGA |


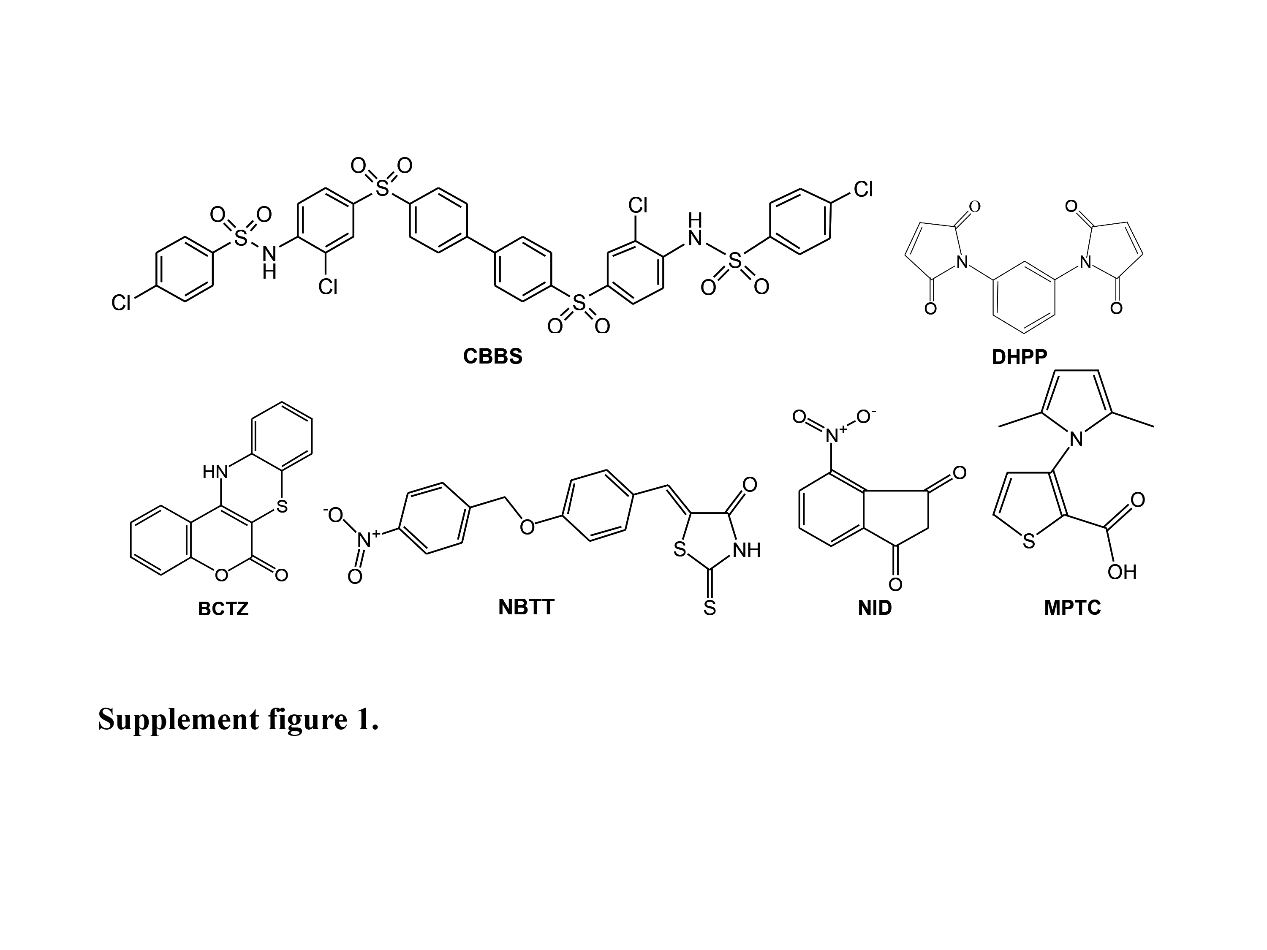
Supplementary figure 1. The molecular structure of different compounds.


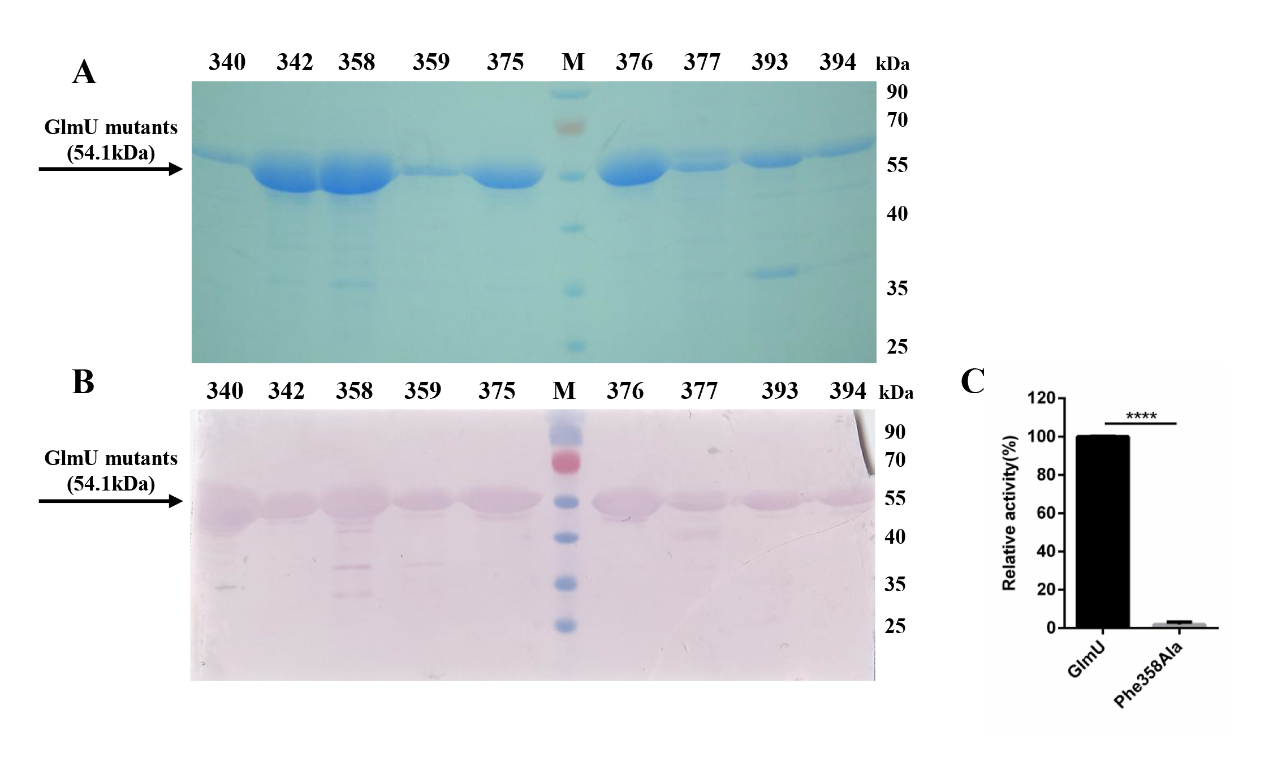
Supplementary figure 2. SDS-PAGE (A), Western blot (B) detection of purified GlmU mutants and the specific activity of GlmU mutant protein Phe358Ala (C). Differences between GlmU and Phe358Ala were calculated by unpaired two-tailed *t*-test. ****, *P* < 0.0001.

M. PageRulerTM Prestained Protein Ladder;

340-394. The purified GlmU mutants with an expected molecular weight of 54.1 kDa.


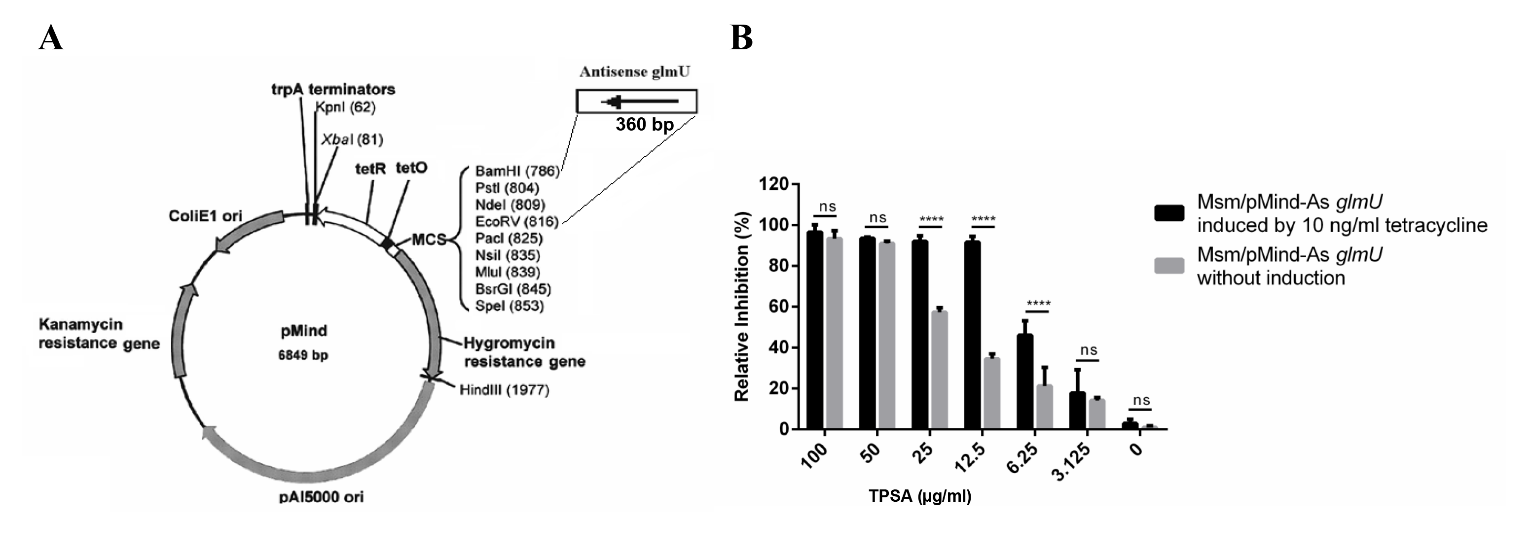


Supplementary figure 3. Map of pMind-As *glmU* (A). Growth inhibition of Msm/pMind-As *glmU* (with 10 ng/ml tetracycline induction and without induction) treated with different concentration TPSA for 24 hours (B). In Supplementary figure 3B, the experiment was performed in triplicates and error bars indicate standard deviation. Differences among groups were calculated by unpaired two-tailed *t*-test. The asterisks represented the statistical differences between the relative inhibition of Msm/pMind-As *glmU* with tetracycline induction and Msm/pMind-As *glmU* without tetracycline induction after treatment by TPSA. ns, no significance; ****, *P* < 0.0001 (100, *P* = 0.4252; 50, *P* = 0.5332; 25, *P* < 0.0001; 12.5, *P* < 0.0001; 6.25, *P* < 0.0001; 3.125, *P* = 0.3473; 0, *P* = 0.6234).


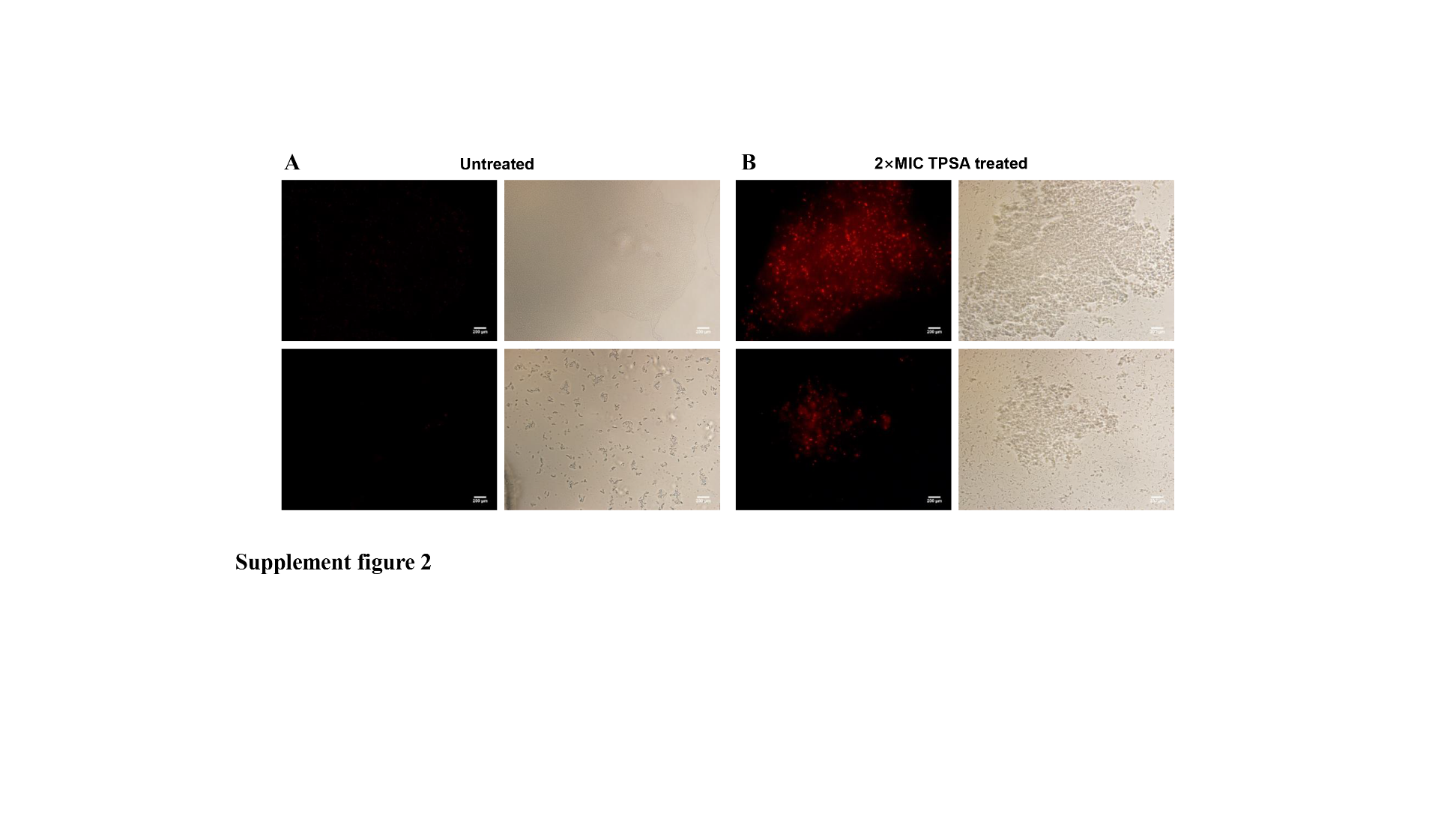
Supplementary figure 4. Fluorescence microscope analysis of bacterial cell wall and membrane integrity by PI uptake. Untreated cells of H37Ra (A) and H37Ra treated with TPSA (2×MIC) for 24h (B). Bacteria cells with compromised cell wall and membrane were permeable to PI and can be observed with red fluorescence.


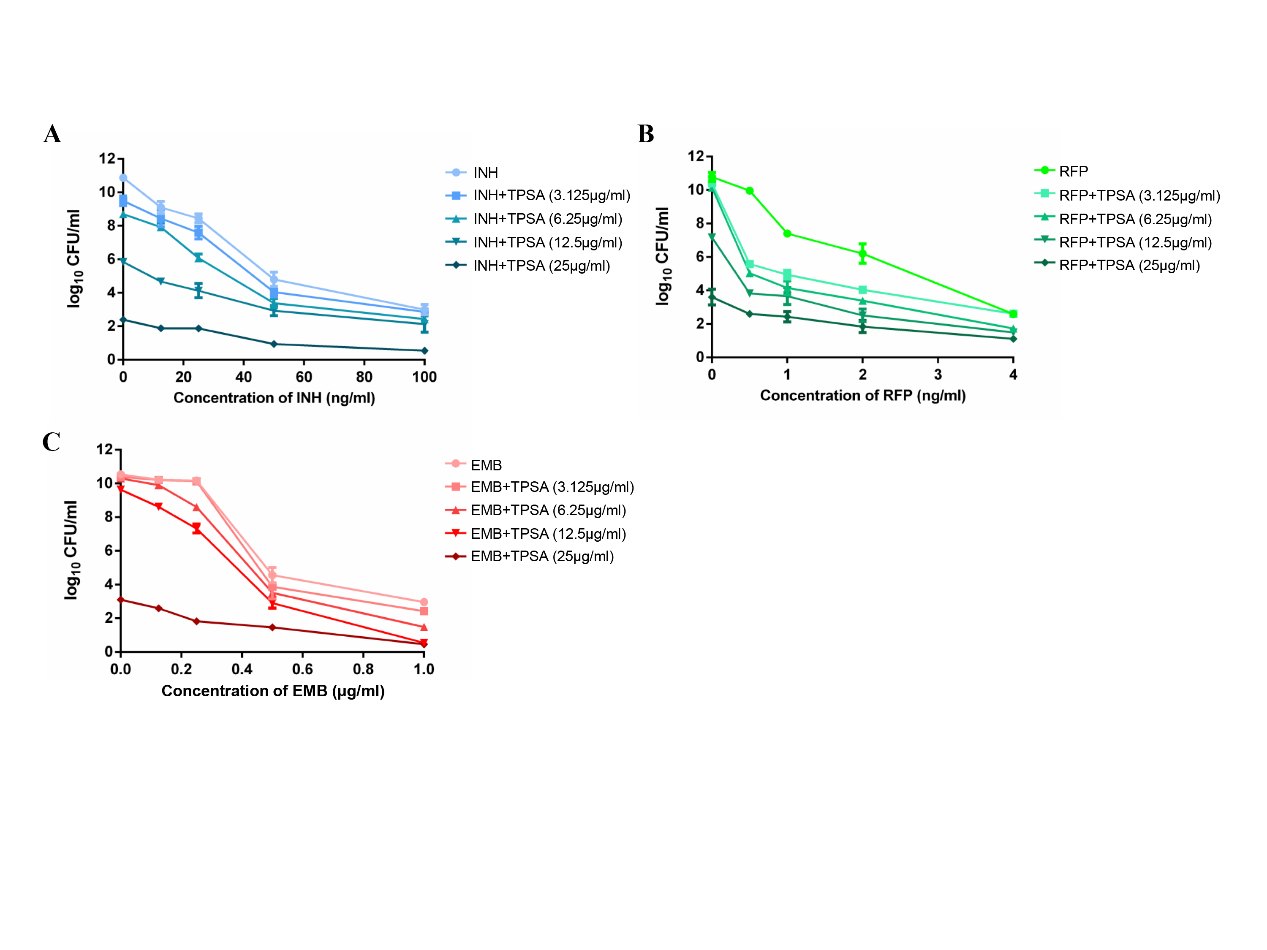
Supplementary figure 5. In vitro bactericidal activity of TPSA and three first-line drugs isoniazid (A), rifampicin (B), and ethambutol (C). Data are representative of one experiment with three independent biological replicates (mean and SD).


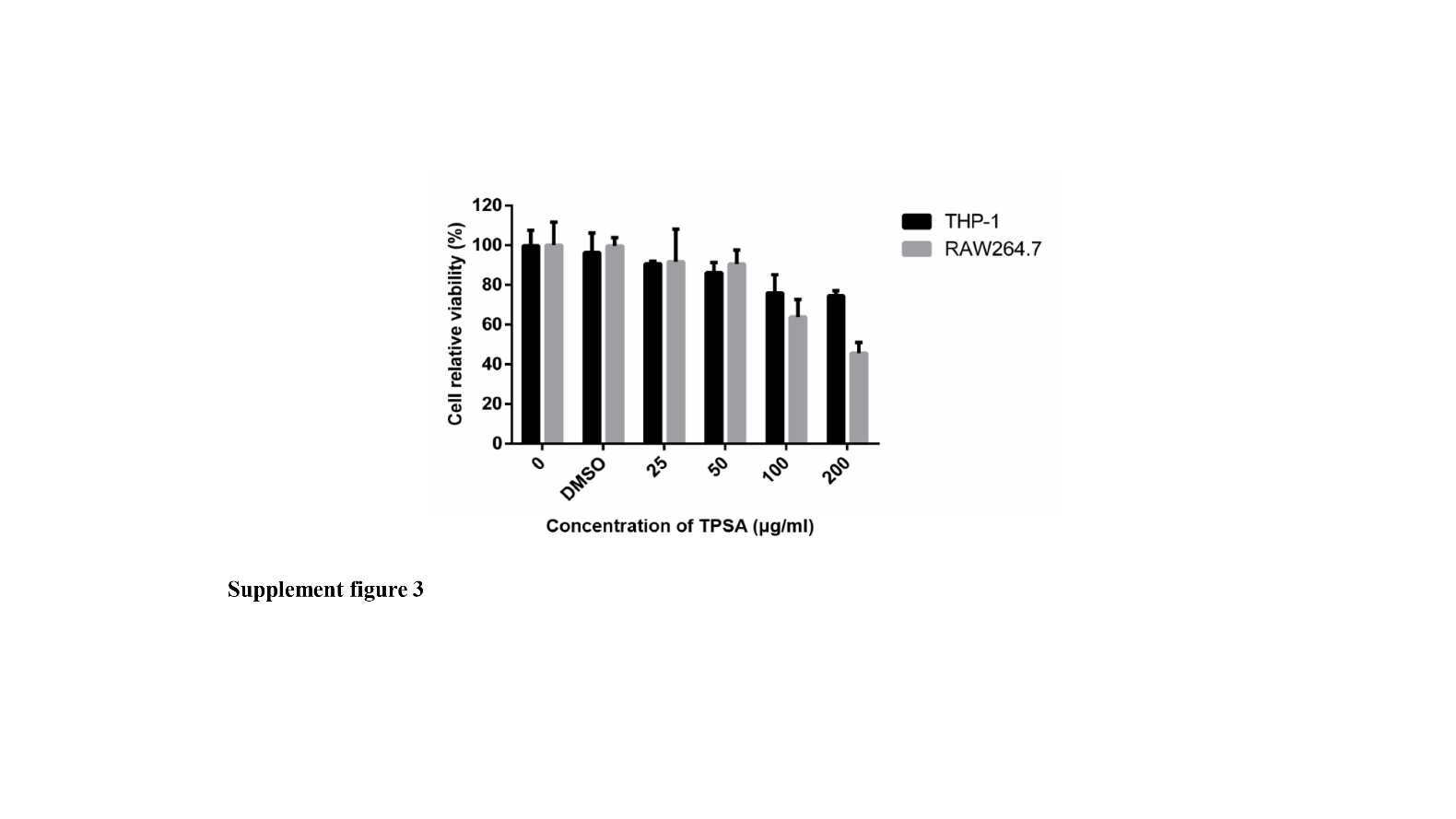
Supplementary figure 6. Cytotoxicity assay of TPSA to macrophage cells. Data are representative of one experiment with three independent biological replicates (mean and SD).


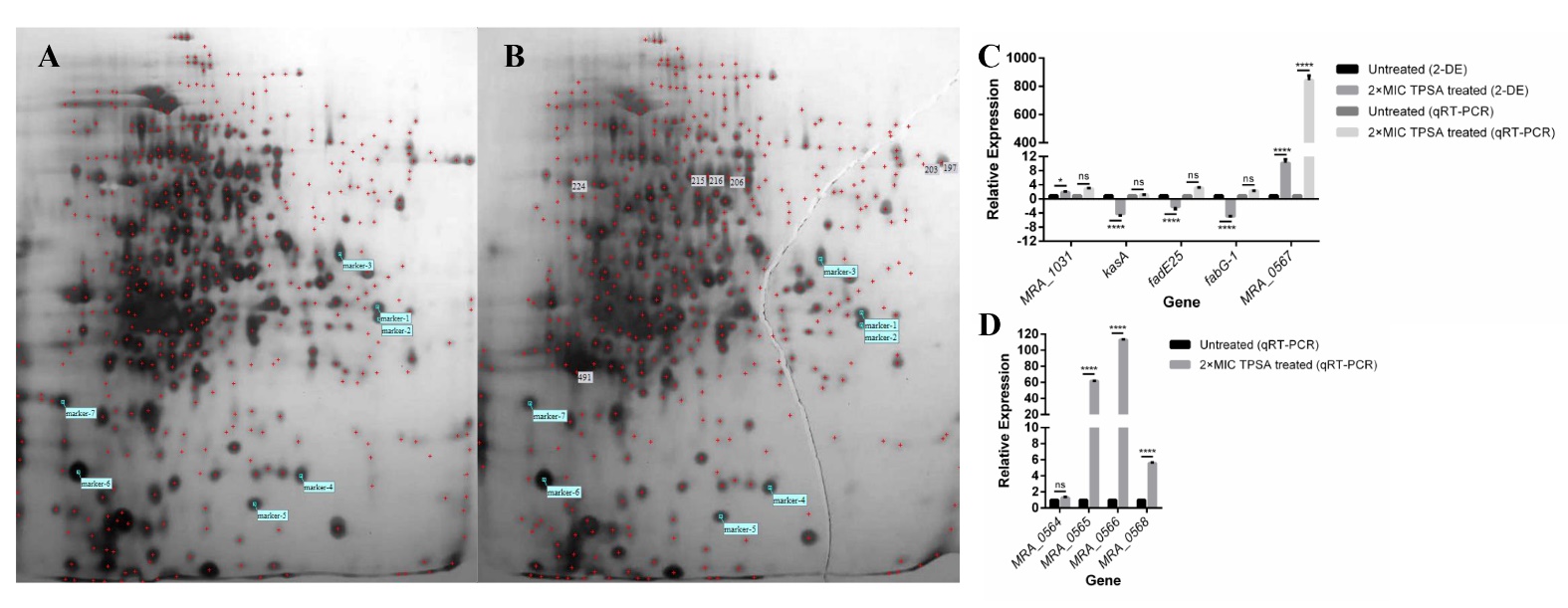
Supplementary figure 7. 2-D electrophoresis of H37Ra in the absence (A) and presence (B) of 50 μg/ml TPSA for 24 hours. Relative change in expression of *MRA_1031*, *kasA*, *fadE25*, *fabG1* and *MRA_0567* in H37Ra treated with 50μg/ml of TPSA for 24 hours (C) and relative change in expression of *MRA_0564*, *MRA_0565*, *MRA_0566* and *MRA_0568* in H37Ra treated with 50μg/ml of TPSA for 24 hours (D). In Supplementary figure 7C and 7D, differences among the groups were calculated by unpaired two-tailed *t*-test. The asterisks represented the statistical differences between relative expression of genes in untreated group and 2×MIC TPSA treated group. ns, no significance; *, *P* < 0.05; ****, *P* < 0.0001 (In Supplementary figure 7C, the *P* values of 2-DE untreated group to 2-DE 2 × MIC TPSA treated group for *MRA_1031*, *P* = 0.0436; *kasA*, *P* < 0.0001; *fadE25*, *P* < 0.0001; *fabG1*, *P* < 0.0001 and *MRA_0567*, *P* < 0.0001. The *P* values of qRT-PCR untreated group to qRT-PCR 2 × MIC TPSA treated group for *MRA_1031*, *P* = 0.8609; *kasA*, *P* = 0.9881; *fadE25*, *P* = 0.8402; *fabG1*, *P* = 0.905 and *MRA_0567*, *P* < 0.0001. In Supplementary figure 7D, the *P* values of untreated group to 2 × MIC TPSA treated group for *MRA_0564*, *P* = 0.2509; *MRA_0565*, *P* < 0.0001; *MRA_0566*, *P* < 0.0001 and *MRA_0568*, *P* < 0.0001).


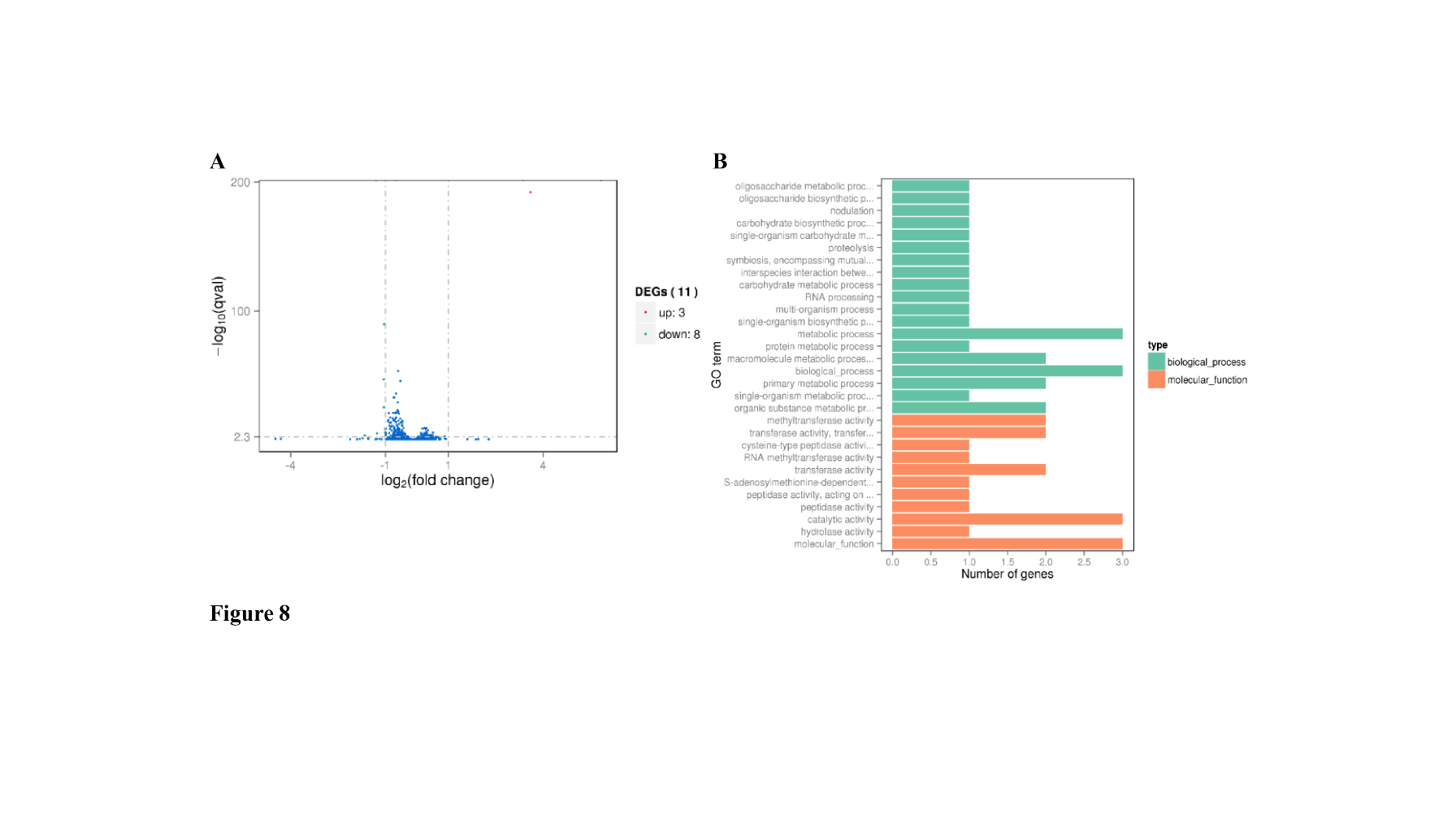
Supplementary figure 8. Differential gene expression of H37Ra treated with TPSA. Volcano plot showed 8 genes with diminished expression and 3 genes with increased expression when treated with TPSA (A). Number of differentially expressed genes by functional categories were shown in enriched GO terms (B).


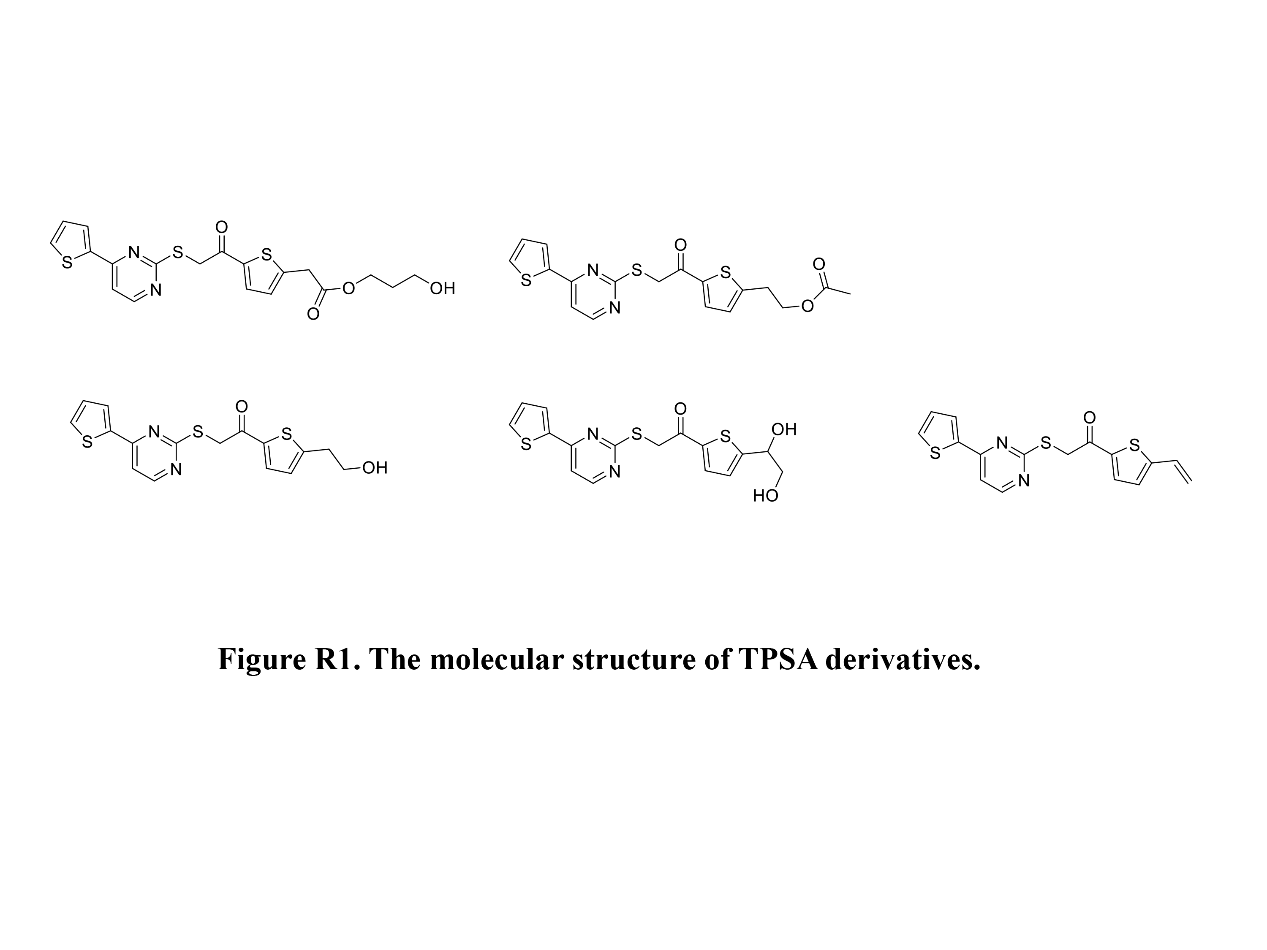
Supplementary figure 9. The molecular structure of TPSA derivatives.
